# Chronic polypharmacy, monotherapy, and deprescribing: Understanding complex effects on the hepatic proteome of aging mice

**DOI:** 10.1111/acel.14357

**Published:** 2024-10-27

**Authors:** Kevin Winardi, John Mach, Matthew J. McKay, Mark P. Molloy, Sarah J. Mitchell, Michael R. MacArthur, Catriona McKenzie, David G. Le Couteur, Sarah N. Hilmer

**Affiliations:** ^1^ Laboratory of Ageing and Pharmacology, Kolling Institute, Faculty of Medicine and Health The University of Sydney and the Northern Sydney Local Health District Sydney New South Wales Australia; ^2^ Bowel Cancer and Biomarker Laboratory, School of Medical Science, Faculty of Medicine and Health University of Sydney Sydney New South Wales Australia; ^3^ Ludwig Princeton Branch Princeton University Princeton New Jersey USA; ^4^ Lewis‐Sigler Institute Princeton University Princeton New Jersey USA; ^5^ Department of Tissue Pathology and Diagnostic Oncology Royal Prince Alfred Hospital Sydney New South Wales Australia; ^6^ Sydney Medical School University of Sydney Sydney New South Wales Australia; ^7^ Charles Perkins Centre University of Sydney Sydney New South Wales Australia; ^8^ ANZAC Research Institute University of Sydney and Concord Hospital Concord New South Wales Australia; ^9^ Centre for Education and Research on Ageing, Faculty of Medicine and Health The University of Sydney Sydney New South Wales Australia

**Keywords:** aging, cholinergic antagonists, deprescribing, drug interactions, liver, mice, polypharmacy, proteome

## Abstract

Polypharmacy (use of ≥5 concurrent medications) is highly prevalent among older adults to manage chronic diseases and is linked to adverse geriatric outcomes, including physical and cognitive functional impairments, falls, frailty, hospitalization, and mortality. Deprescribing (withdrawal) is a potential strategy to manage polypharmacy. The broad molecular changes by which polypharmacy causes harm and deprescribing may be beneficial are unknown and unfeasible to study rigorously in tissue from geriatric patients. Therefore, in a randomized controlled trial, we administered therapeutic doses of commonly used chronic medications (oxycodone, oxybutynin, citalopram, simvastatin, or metoprolol) as monotherapy or concurrently (polypharmacy) from middle‐age (12 months) to old‐age (26 months) to male C57BL/6J (B6) mice and deprescribed (gradually withdrew) treatments in a subset from age 21 months. We compared drug‐related hepatic effects by applying proteomics along with transcriptomics and histology. We found that monotherapy effects on hepatic proteomics were limited but significant changes were seen with polypharmacy (93% unique to polypharmacy). Polypharmacy altered the hepatic expression of proteins involved in immunity, and in drug, cholesterol, and amino acid metabolism, accompanied by higher serum drug levels than monotherapies. Deprescribing not only reversed some effects but also caused irreversible and novel changes in the hepatic proteome. Furthermore, our study identified several hepatic protein co‐expressed modules that are associated with clinically relevant adverse geriatric outcomes, such as mobility, frailty, and activities of daily living. This study highlights the complex molecular changes following aging, chronic polypharmacy, and deprescribing. Further exploration of these mechanistic pathways may inform management of polypharmacy and deprescribing in older adults.

## Graphical abstract

Chronic polypharmacy (use of five or more medications) is common in older adults, is associated with adverse outcomes, and can be managed with deprescribing (cessation of medication). We investigated the effect of chronic polypharmacy and the constituent monotherapies on the liver proteome, transcriptome, histology, and serum drug levels in aged mice. Polypharmacy substantially altered the expression of proteins involved in drug metabolism, immunity, cholesterol metabolism, and amino acid metabolism to a greater extent than the constituent monotherapies, while deprescribing reversed some, but not all, changes and induced novel protein expression changes.

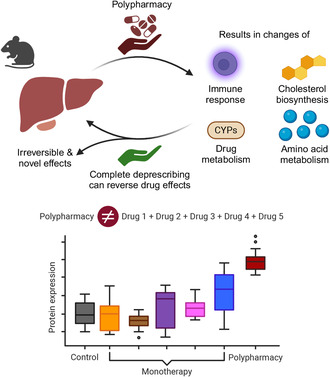

**Abbreviations**
α‐SMA: alpha smooth muscle actin
CI: credible interval
CYP: cytochrome P450
DMET: drug‐metabolizing enzyme and transporter
FC: fold change
FDR: false discovery rate
GO: gene ontology
GS: gene significance
GSEA: gene set enrichment analysis
H&E: hematoxylin and eosin
HCA: hierarchical clustering analysis
KEGG: Kyoto encyclopedia of genes and genomes
LC‐MS/MS: liquid chromatography‐tandem mass spectrometry
MGI GXD: Mouse Genome Informatics Gene eXpression Database
MHC: Major histocompatibility complex
MM: module membership
MS: mass spectrometry
ORA: over‐representation analysis
PCA: principal component analysis
PDI: protein‐drug interaction
RNA‐seq: bulk RNA sequencing
STITCH: Search Tool for Interacting Chemicals
STRING: Search Tool for the Retrieval of Interacting Genes/Proteins
TOM: topological overlap matrix
TPM: transcript per million
WGCNA: weighted gene co‐expression network analysis

## 1 INTRODUCTION

Old age is accompanied by an increase in multimorbidity (Barnett et al., 2012), which is often managed with complex combinations of medications. Polypharmacy, the concurrent use of five or more medications (Gnjidic et al., 2012), currently affects one‐third to two‐thirds of older adults globally (Page et al., 2019; Wastesson et al., 2018). Its prevalence is rising as the population ages and advances in research and healthcare deliver more medications. The current models of drug development, evaluation, and health care, focused on improving outcomes in a single disease, do not address the needs of older adults with multimorbidity and polypharmacy (Liu et al., 2022). Polypharmacy is associated with poor outcomes such as impaired physical and cognitive function (Aljeaidi & Tan, 2022; Rawle et al., 2018), increased falls (Zaninotto et al., 2020), frailty (Arauna et al., 2020; Setiati et al., 2021), hospitalization (Zaninotto et al., 2020), and mortality (Li et al., 2022). For example, approximately 16.5% of acute hospital admissions in a UK hospital were attributable to adverse drug reactions, which predominantly occurred in older adults with multimorbidity and polypharmacy (Rostam et al., 2022). Due to its mounting worldwide prevalence and well‐documented risks, polypharmacy is a priority area of the WHO initiative: *Medication without Harm* (Donaldson et al., 2017). Best practice for management of polypharmacy includes medication review and deprescribing (the clinical process of discontinuing medications where harm outweighs benefit) with shared decision‐making (Scott et al., 2015). However, informed shared decision‐making is limited by the lack of understanding of the mechanisms of the effects of polypharmacy and of deprescribing. This mechanistic knowledge will provide better‐informed predictions to optimize prescribing and deprescribing in patients with polypharmacy.

There is very little cellular and molecular data to guide prescribing in polypharmacy or deprescribing, particularly in regard to how multiple concurrent drugs interact, effects of chronic therapy and their reversibility, and what this may mean for geriatric outcomes. The liver orchestrates several essential homeostatic processes, including drug/xenobiotic metabolism, immune response, and cholesterol, vitamin, bile, glucose, and protein metabolism (Ben‐Moshe & Itzkovitz, 2019). Most drugs are metabolized by cytochrome P450 enzymes (CYPs) (Willson & Kliewer, 2002), and their activity is known to change with aging (Kwak et al., 2015; Zidong Donna et al., 2012) and with medication use (Herrlinger & Klotz, 2001; Willson & Kliewer, 2002). Current understanding of drug–drug interactions is generally based on drug pairs, which has limited utility when considering the complicated case of polypharmacy. At the molecular level, it is poorly understood how drug use or reducing drugs may have long‐term alterations on the hepatic proteome, particularly as a function of age, sex, and chronic treatment. The development of omics technology and systems biology opens the possibility to explore the effect of interventions on cellular and molecular dynamics, identify complex protein–drug interactions, and discover molecular biomarkers associated with functional outcomes.

As it is not ethical or feasible to conduct interventional studies and source human tissue, we developed a murine model of chronic polypharmacy, monotherapy, and deprescribing with medications commonly used in older adults (Mach, Gemikonakli, et al., 2021). This murine model displayed outcomes comparable to those observed in older patients wherein polypharmacy increased frailty and decreased physical function, with some outcomes reversed by deprescribing. Here, we explored the hepatic molecular signature following chronic monotherapy, polypharmacy, and deprescribing in aging mice. Our approach encompassed an exploration of the global hepatic proteome alterations induced by chronic pharmacotherapies, followed by a comparative analysis between the monotherapies and polypharmacy signatures. We found that polypharmacy induced hepatic proteome changes to a greater extent than the monotherapies. Next, we specifically probed for pharmacologically relevant proteins and explored differences in serum drug levels. We observed that polypharmacy induced major changes in pharmacokinetics‐related proteins and had higher serum drug levels compared with the corresponding monotherapies. We also compared the molecular effects of deprescribing with the drug‐related effects. We discovered that with deprescribing, some drug‐related changes are reversible, others are irreversible and some novel changes occur in the hepatic proteome. Finally, we applied a systems pharmacology approach to explore the relationship of clinically relevant phenotypes with the hepatic proteome reorganization.

## 2 RESULTS

**FIGURE 1:**
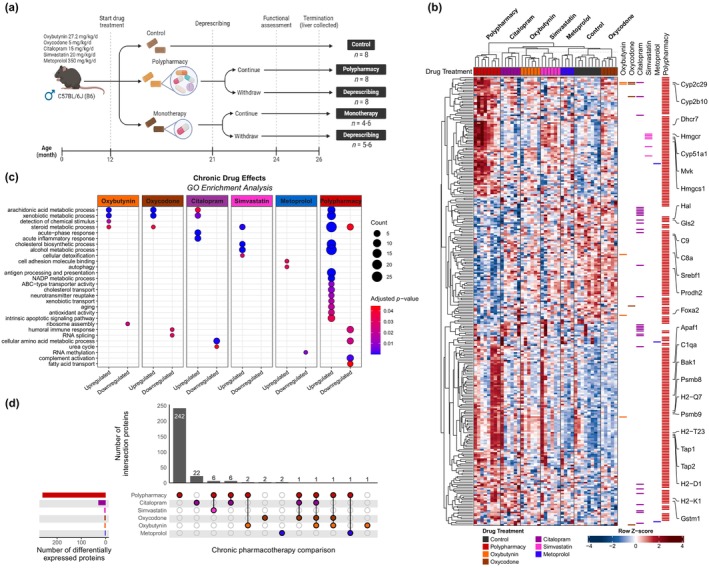
Hepatic proteome signatures of chronic monotherapy and polypharmacy. (a) Healthy male C57BL/6J mice were aged until they reached 12 months old before randomization into drug treatment allocation to receive either control, monotherapy, or polypharmacy. The monotherapy feed contains only one of the five medications in the polypharmacy regimen at the same therapeutic dose. The mice underwent drug treatment until the age of 21 months when they were re‐randomized to either continue treatment or to deprescribe all medications. Deprescribing occurred over the course of 6 weeks. Overall, this amounts to 13 different treatment groups: Control, six chronic (continuous) drug treatment groups (polypharmacy and five monotherapies), and six corresponding deprescribing groups. This study applied (functional) behavioral data at the age of 24 months. At the age of 26 months, the mice were terminated and livers were collected. Sample sizes for hepatic proteomics analysis: Control, *n* = 8; oxybutynin, *n* = 6; oxycodone, *n* = 5; citalopram, *n* = 6, simvastatin, *n* = 6; metoprolol, *n* = 4; polypharmacy, *n* = 8; oxybutynin deprescribed, *n* = 6; oxycodone deprescribed, *n* = 5; citalopram deprescribed, *n* = 6, simvastatin deprescribed, *n* = 6; metoprolol deprescribed, *n* = 5; polypharmacy deprescribed, *n* = 8. (b) Heatmap after semi‐supervised hierarchical clustering of all differentially expressed proteins across the chronic monotherapies and polypharmacy against control animals (false discovery rate [FDR]‐adjusted *p* < 0.10; fold change [FC] > ±1.50). Hierarchical clustering was conducted with Euclidean distance and complete linkage. Column clustering was performed with predetermined treatment allocation whereas row clustering was unsupervised. Heatmap is color‐coded with row *z*‐score scaling with red indicating higher abundance and blue indicating lower. Proteins that were significantly different comparing chronic drug versus control pairwise comparison are annotated as bars on the right of the heatmap within their corresponding drug column. (c) Dot plot showing over‐representation analysis of selected gene ontology (GO) pathways enriched in chronic drug treatment compared with control, with size of dots proportional to protein ratio and colors representing FDR‐adjusted *p*‐value. (d) The number of shared (overlapping) and unique differentially expressed proteins across treatment groups are recorded within an UpSet plot. The total number of differentially expressed proteins in each chronic drug treatment was noted as the horizontal bar plot on the left.

To investigate the molecular effects of monotherapy, polypharmacy, and deprescribing, we carried out proteomic analysis on the liver of the chronic polypharmacy mouse model we have developed (Mach, Gemikonakli, et al., 2021) (Figure 1a). Briefly, healthy adult male mice treated chronically from 12 months of age, with either standard chow (control), polypharmacy feed, or one of the five different constituent monotherapies. The drugs within the polypharmacy regimen were therapeutic doses of oxybutynin (anticholinergic for urinary incontinence), oxycodone (opioid analgesic), citalopram (selective serotonin reuptake inhibitor antidepressant), simvastatin (cholesterol‐lowering agent), and metoprolol (β‐blocker antihypertensive). Drugs used in the model were selected because they are commonly used chronically by older adults and have similar pharmacokinetics and pharmacodynamics in mice and humans, with an over‐representation of drugs with anticholinergic or sedative effects (i.e., oxybutynin, oxycodone, and citalopram) because these drugs are particularly associated with adverse geriatric outcomes (Liu et al., 2024; Mehdizadeh et al., 2021; Taylor‐Rowan et al., 2021). After 9 months of treatment at 21 months of age, each drug‐treated group of mice was re‐randomized to either continue drug treatment or to gradually, completely deprescribe (withdraw) the medications, with all drugs completely withdrawn by age 23 months. At 26 months of age, livers were collected and analyzed for mass spectrometry (MS)‐based proteomics. The use of medications from age 12 to 26 months in mice models human treatment from middle age (35–45) to old age (65–75 years). A total of 6489 proteins from the livers of 79 individual animals (*n* = 4–8/group) across the 13 treatment groups were analyzed (Figure S1, Table S1, see methods).

### 2.1 Chronic monotherapy caused minimal effects on the hepatic proteome but polypharmacy caused many changes

We first explored the effects of chronic drug treatment of mice from the age of 12 months to 26 months, representing middle to old age. Across the chronic monotherapies, we observed a limited effect on hepatic protein expression compared with control animals: oxybutynin (3 up‐ and 2 downregulated), oxycodone (3 up‐ and 2 downregulated), citalopram (17 up‐ and 13 downregulated), simvastatin (6 upregulated), and metoprolol (2 up‐ and 1 downregulated) (false discovery rate [FDR]‐adjusted *p* < 0.10; fold change [FC] > ±1.50) (Figure 1b–d; Table S2). Consistently, principal component analysis (PCA) of the hepatic proteomes did not reveal a clear separation between the chronic monotherapies and control, indicating only minor effects (Figure S2a). The monotherapies affected a variety of proteins with different functions (Figure 1c; Table S3). Most interestingly, chronic oxybutynin monotherapy increased the expressions of Cyp2c29 and Cyp2b10 by 1.97‐ and 7.99‐fold, respectively, while also altering the expressions of a receptor chaperone (Rtp4), a mitochondrial GTPase (Eral1), and an apoptotic regulator (Caap1). The same two CYP enzymes were also upregulated in response to chronic oxycodone monotherapy, to a greater extent (2.03‐ and 14.36‐fold for Cyp2c29 and Cyp2b10, respectively). Oxycodone also increased Cyp2c55 expression by 2.29‐fold and reduced the expressions of ribosomal subunit (Fau) and RNA‐binding protein (Rbm42) compared with control mice. Citalopram induced the most changes with a total of 30 differentially expressed proteins, including several CYPs (Cyp2c55, Cyp2c29, and Cyp2c70), carboxylesterase 3B (Ces3b), amino acid metabolic enzymes (Gls2, Cps1, Afmid, and Acmsd), and several inflammatory proteins (Itih4, Lbp, Hp, and Orm1). Livers from simvastatin monotherapy mice expressed higher levels of cholesterol biosynthetic proteins (Cyp51a1 and Mvk) along with its primary drug target (Endo et al., 1976), HMG‐CoA reductase (Hmgcr), in accordance with murine (Schonewille et al., 2016) and cell‐based (Mitchell et al., 2023) studies. Simvastatin also upregulated cholesterol regulatory protein (Tmem97), retinol dehydrogenase (Rdh11), and annexin 13 (Anx13). Finally, metoprolol increased the expression of cathepsin D (Ctsd) and an adhesion protein (Bcam), and decreased mitochondrial methyltransferase (Mettl15). Gene set enrichment analysis (GSEA) revealed non‐specific drug effects, wherein monotherapies independently altered the hepatic proteome in recurring themes, affecting proteins related to immune functions, lipid metabolism, amino acid metabolism, and mitochondrial processes, among others (Figure S2b; Table S4). Overall, chronic monotherapy administered at a therapeutic dose had minimal impact on the healthy aged hepatic proteome.

It has been assumed that the effects of polypharmacy reflect the additive effects of each drug administered, and therefore, we expected only minor changes in the hepatic proteome with polypharmacy. However, we observed 260 proteins with altered expression following chronic polypharmacy compared with control (FDR‐adjusted *p* < 0.10; FC > ±1.50), with 189 up‐ and 71 downregulated proteins (Figure 1b–d; Table S2). Within the PCA, polypharmacy was partially separated from control by PC1 (Figure S2a). Polypharmacy expressed higher levels of cholesterol biosynthetic proteins (Hmgcr, Mvk, Dhcr7, Fdps, and Hmgcs1), drug‐metabolizing enzymes (more details in section 2.3) and immune response proteins (Stat1, Ifit3, Tap1, and H2‐D1), while downregulating amino acid metabolic enzymes (Hal, Gls2, and Prodh2), among others (Figures 1c and S2b; Tables S3 and S4). Polypharmacy also altered the expressions of the complement system effectors (C1qa, C8a, C9, and Cfh), apoptotic proteins (Apaf1 and Bak1), mitochondrial proteins (Atp5if1 and Cox7a2l), proteosomal subunits (Psmb8 and Psmb9), cathepsin proteases (Ctsc, Ctsd, and Ctss) along with transcriptional factors (Foxa2 and Srebf1). Predictably, a high degree of overlap between polypharmacy and the monotherapies was observed (Figure 1d). Specifically, 80% (four out of five proteins) were shared between oxybutynin monotherapy and polypharmacy, 60% (3/5) for oxycodone, 27% (8/30) for citalopram, 100% (6/6) for simvastatin, and 33% (1/3) for metoprolol. Since monotherapies altogether differentially expressed only 45 unique proteins, of the 260 differentially expressed proteins in polypharmacy, 242 (93.08%) were unique to polypharmacy, demonstrating that exposure to multiple drugs chronically and concurrently induced novel and pronounced changes in the liver.

### 2.2 Monotherapy drug effects were reflected in the polypharmacy signature along with synergistic effects

**FIGURE 2:**
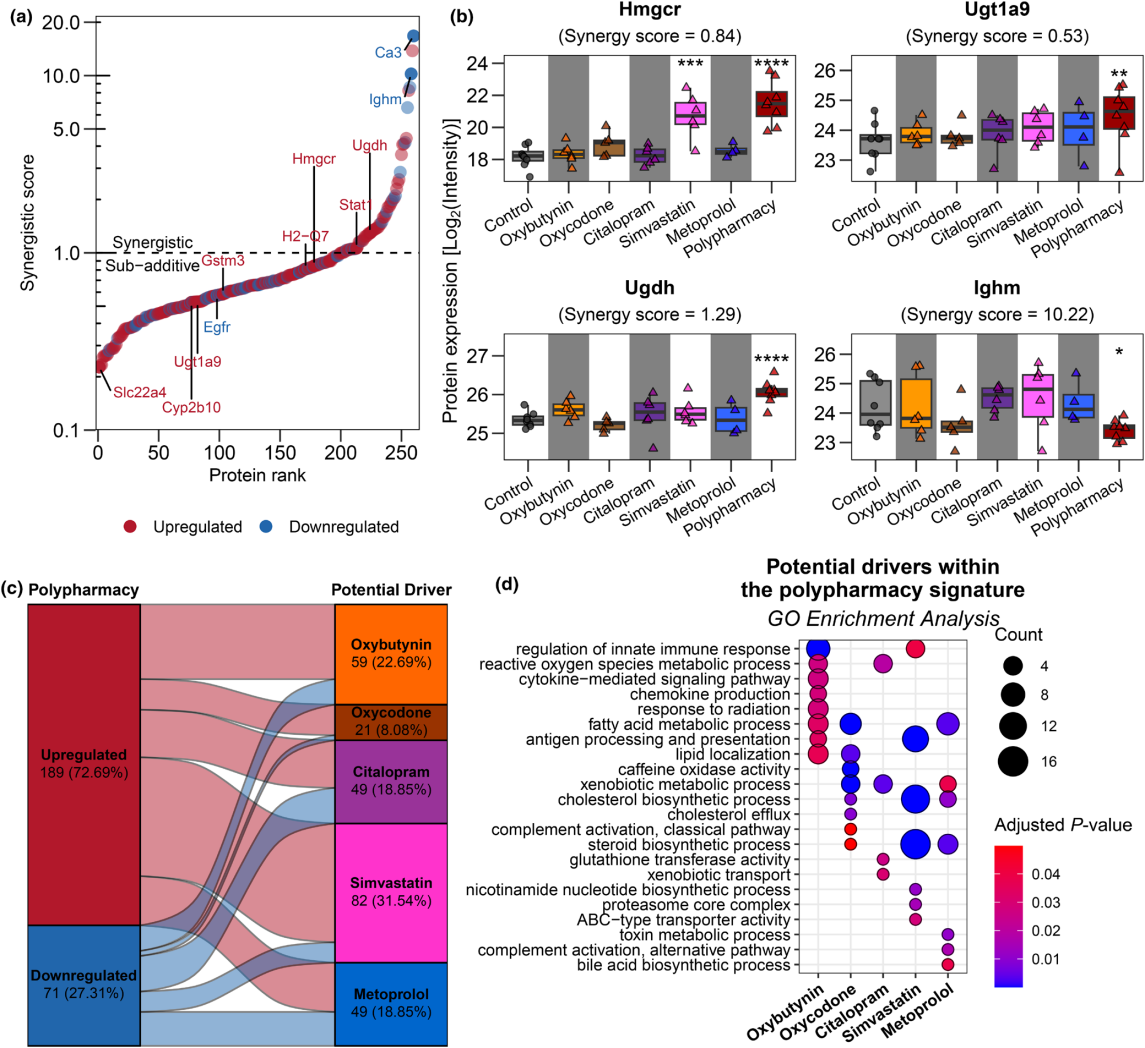
Comparing hepatic signatures of chronic monotherapy and polypharmacy. (a) Drug synergism score across the 260 differentially expressed proteins in the polypharmacy signature. Horizontal dotted line at score = 1.00 indicates threshold that determines synergistic (>1.00) or sub‐additive effects (≤1.00). (b) Box plots of Hmgcr, Ugt1a9, Ugdh, and Ighm. Synergistic score was annotated. Differential expression result comparing chronic pharmacotherapy against control; * False discovery rate (FDR)‐adjusted *p* < 0.10, **p < 0.05, ***p < 0.01, ****p < 0.001. (c) Alluvial plot that depicts the single‐most dominant drug driver of each protein within the polypharmacy signature, creating five drug clusters with varying contributions. Protein counts and proportion (%) are shown. (d) Dot plot showing selected gene ontology (GO) pathways enriched in the five “dominant drug” clusters within the polypharmacy signature, with size of dots proportional to protein counts and colors representing FDR‐adjusted *p*‐value.

Given the striking difference between chronic monotherapy and polypharmacy signatures, we adopted principles from drug interaction studies to more comprehensively evaluate whether the effects observed in polypharmacy were genuinely novel or whether monotherapies can predict those changes. We applied a modified omics version of the response additivity model formulated by Megger et al. (2018) (see methods) and found that most proteins in the polypharmacy signature were sub‐additively regulated (score ≤1.00; 200/260; 76.92%) (Figure 2a; Table S5), whereby the sum of monotherapies was greater than the polypharmacy effect. There are multiple different patterns of sub‐additive polypharmacy effect. In some instances, one or a few monotherapies drives the expression in polypharmacy, as seen in Hmgcr (Figure 2b). In other cases such as Ugt1a9 (Figure 2b), individual monotherapies are not sufficient to produce a significant effect, but have directional concordance such that a significant and pronounced effect is only observed in polypharmacy. The remaining 60 proteins were synergistically regulated (score >1.00; 60/260; 23.08%) (Figure 2a). UDP‐glucose 6‐dehydrogenase (Ugdh) was marginally synergistically regulated, whereby polypharmacy altered the expression more than the sum of the monotherapy‐induced changes (Figure 2b). Interestingly, most synergism arose through non‐canonical mechanisms where different drug effects were in different directions. For example, immunoglobulin heavy constant Mu (Ighm) had a high synergistic score of 10.22, because the expression was altered differently across monotherapies, and therefore, the sum monotherapy effect was −0.08 (i.e., no change in polypharmacy signature was expected) (Figure 2b). However, the observed polypharmacy Ighm profile was most similar to the oxycodone signature, suggesting that oxycodone may be the dominant driver of this change.

Observations like the Ighm profile prompted us to identify the drug that potentially caused the alterations in the polypharmacy signature. To achieve this, we identified the monotherapy with the most similar protein change to that of polypharmacy (Table S5). We observed disproportionate contributions from each drug to the polypharmacy signature, in which 59, 21, 49, 82, and 49 proteins within the polypharmacy signature were potentially driven by oxybutynin (22.69%), oxycodone (8.08%), citalopram (18.85%), simvastatin (31.54%), and metoprolol (18.85%), respectively (Figure 2c). Polypharmacy‐induced changes in drug‐metabolizing enzymes were driven by oxycodone, citalopram, and metoprolol (Figure 2d; Table S6). Immune‐related and complement system effectors were potentially influenced by oxybutynin, oxycodone, and metoprolol, whereas cholesterol biosynthetic proteins were primarily driven by simvastatin, but oxycodone and metoprolol also contributed (Figure 2d; Table S6). Following that, we applied a similar analysis with a focus on synergism, which yielded six clusters: five drug clusters and one synergistic cluster (Figure S3a). This synergistic polypharmacy cluster consisted of immune‐related and cholesterol metabolic proteins (Figure S3b; Table S7). While drug clusters remained relatively similar in terms of enriched pathways, there are noticeable differences where enrichment shifts to the synergistic cluster (Figure S3b; Table S7). For example, regulation of innate immune response and classical complement activation pathways were from simvastatin and oxycodone, respectively. These findings indicate a complex interplay among medications, whereby chronic monotherapy caused minor effects on the hepatic proteome that were substantially induced by polypharmacy, with differential expression governed either by a single drug or by multiple drugs.

### 2.3 Pharmacokinetics‐ and pharmacodynamics‐related hepatic proteins were altered in polypharmacy and some monotherapies

**FIGURE 3:**
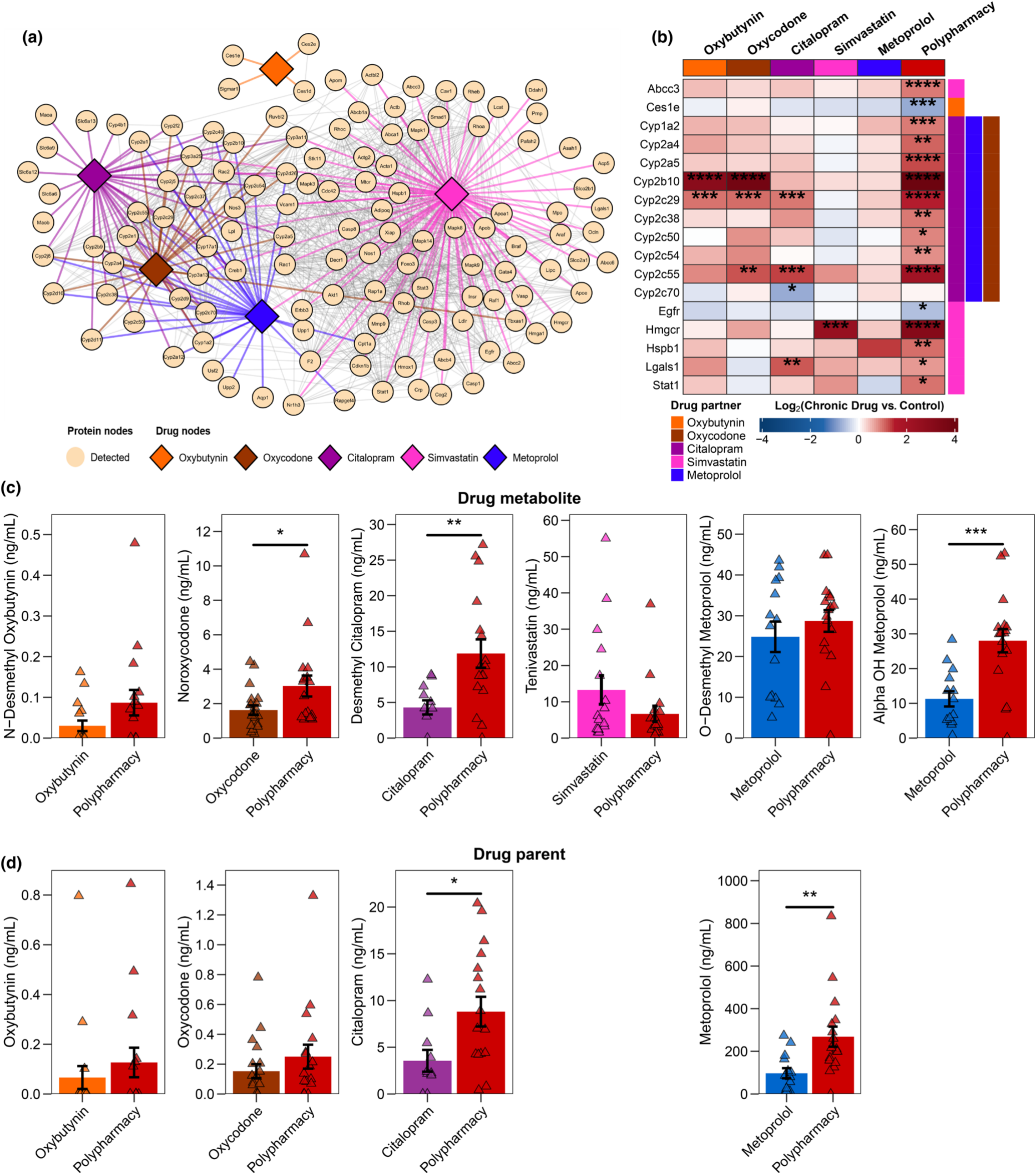
Probing for pharmacologically relevant proteins. (a) Protein–drug interaction network derived from Search Tool for Interacting Chemicals (STITCH) database with protein nodes (circle), drug nodes (rhombus), and their interactions color‐coded accordingly. Protein–protein interaction edges are in gray. Only proteins that were quantified in our proteomics dataset (*n* = 123) are depicted in the network (full network in Figure S4a). (b) Heatmap summarizing differential expression analysis results of pharmacologically relevant proteins. Only proteins with significant change comparing chronic drug treatment against control were depicted; * False discovery rate (FDR)‐adjusted *p* < 0.10, **p < 0.05, ***p < 0.01, ****p < 0.001. The known drug interactor with the proteins was annotated by the colored bar on the right‐hand side column of the heatmap. Serum levels of (c) drug metabolites and (d) drug parents from 24‐month‐old mice comparing polypharmacy (*n* = 16) against the corresponding monotherapies (oxybutynin [*n* = 18], oxycodone [*n* = 19], citalopram [*n* = 11], simvastatin [*n* = 16], and metoprolol [*n* = 14]). Six drug metabolites were quantified (one for each drug and two for metoprolol), while only four parent drug molecules were successfully quantified. Data are represented as mean ± SEM. Two‐tailed Student's *t* test was conducted and significance was recorded: *p < 0.05, **p < 0.01, ***p < 0.001.

To gain mechanistic insights into drug‐related effects, we investigated proteins with an established direct protein–drug interaction extracted from the Search Tool for Interacting Chemicals (STITCH) database (Szklarczyk et al., 2016). These pharmacologically relevant proteins include drug‐metabolizing enzymes, transporters, and drug targets. We identified 361 pharmacologically relevant proteins across the five drugs, with simvastatin having the most known interactions (Figure S4a,b). Of these, 123 were quantified in our proteomics dataset (Figure 3a): 118 of the 216 proteins known to be expressed in the liver and five additional proteins (Acta1, Actbl2, Adipoq, Mpo, and Nos1) (Table S8; Figure S4a,c).

The most commonly altered pharmacologically relevant hepatic proteins were the CYP drug‐metabolizing enzymes (FDR‐adjusted *p* < 0.10; FC > ±1.50; Figure 3b). Several CYP enzymes (e.g., Cyp2b10 and Cyp2c29) exhibited upregulation in both polypharmacy and certain monotherapies, whereas other CYPs (e.g., Cyp1a2 and Cyp2a5) were exclusively altered in polypharmacy (Figure 3b). These CYP enzymes are known to interact with citalopram, metoprolol, and oxycodone. Other pharmacokinetics‐related proteins, including carboxylesterase 1E (Ces1e; mediates oxybutynin hydrolysis; Yuichiro et al., 2012) and ATP‐binding cassette transporter 3 (Abcc3; statin efflux transporter; Feng et al., 2021) were also altered (Figure 3b). Additionally, we also manually probed our proteomics dataset for drug‐metabolizing enzymes and transporters (DMETs) beyond those identified from STITCH, and a total of 117 drug‐metabolizing enzymes across 11 classes and 141 transporters across two classes were identified (Figure S5). Polypharmacy also altered the expression of several phase II drug‐metabolizing enzymes, including UDP‐glucuronosyltransferases (e.g., Ugt1a2 and Ugt2a1), glutathione S‐transferases (e.g., Gstm1, Gstm2, and Gstm3), and NAD(P)H quinone dehydrogenase 1 (Nqo1) along with several solute carrier transporters (e.g., Slc22a1 and Slc22a4) (Figure S5).

Altered expression of drug metabolizing enzyme and transporter proteins (DMETs) may have pharmacokinetics implications. To assess that, we next analyzed serum drug parent and metabolite levels at 24 months of age. Even though doses received were the same in polypharmacy and the corresponding monotherapies, we observed major differences in serum drug levels. With serum metabolites, polypharmacy consistently had higher serum levels of noroxycodone, desmethyl citalopram, and α‐hydroxyl metoprolol compared with the corresponding monotherapy (*p* < 0.05) (Figure 3c). This highlights that the increase in DMETs may be an adaptive response to chronic drug exposure to increase the rate of drug metabolism. However, polypharmacy concurrently had higher serum levels of parent drug species for citalopram and metoprolol compared with the respective monotherapies (*p* < 0.05) (Figure 3d). Interestingly, the metabolite‐to‐parent ratios were not different between the monotherapies and polypharmacy (Figure S4d). Altogether, despite having increased hepatic DMET expressions, polypharmacy is characterized by a higher drug exposure compared with monotherapies.

In addition to pharmacokinetics‐related proteins, we observed alterations with pharmacodynamics‐related and aging‐related proteins (FDR‐adjusted *p* < 0.10; FC > ±1.50). The protein target of simvastatin (Endo et al., 1976), Hmgcr, was upregulated in polypharmacy and simvastatin monotherapy by 8.94‐ and 5.73‐fold, respectively (Figure 3b). Polypharmacy alone also upregulated epidermal growth factor receptor (Egfr), heat shock protein β1 (Hspb1), galectin‐1 (Lgals1), and signal transducer and activator of transcription 1 (Stat1) by 1.51‐, 1.69‐, 1.52‐, and 1.97‐fold, respectively (Figure 3b). All of those proteins are implicated in aging and inflammation (Kamat et al., 2008; Kiss et al., 2023; Omrani et al., 2023; Rasa et al., 2022; Schultz et al., 2001), and are also modulated by statins in other experimental models (Koike et al., 2021; Nègre‐Aminou et al., 2002; Pereira et al., 2020). Lgals1 was also upregulated in citalopram monotherapy. Interestingly, with both polypharmacy and monotherapy, we did not observe any protein changes related to the cholinergic, serotonergic, and adrenergic neurotransmission system. We did observe changes in Slc22a4 (also known as organic cation transporter 1 [OCT1]) has been reported to interact with oxybutynin and the metabolite of oxycodone (noroxycodone) and also transports neurotransmitters including serotonin (Zhou et al., 2021).

### 2.4 Deprescribing can reverse molecular signatures of chronic pharmacotherapy

Preclinical (Mach, Gemikonakli, et al., 2021) and clinical (Ibrahim et al., 2021) studies suggest that deprescribing may alleviate drug‐associated adverse geriatric outcomes; however, molecular insights are still lacking. Here, we also investigated the molecular reversibility of complete medication deprescribing, with drugs gradually and completely withdrawn over up to 6 weeks, and livers collected at least 18 weeks after complete cessation. PCA revealed that deprescribing produced varied hepatic proteome profiles (Figure S6).

**FIGURE 4:**
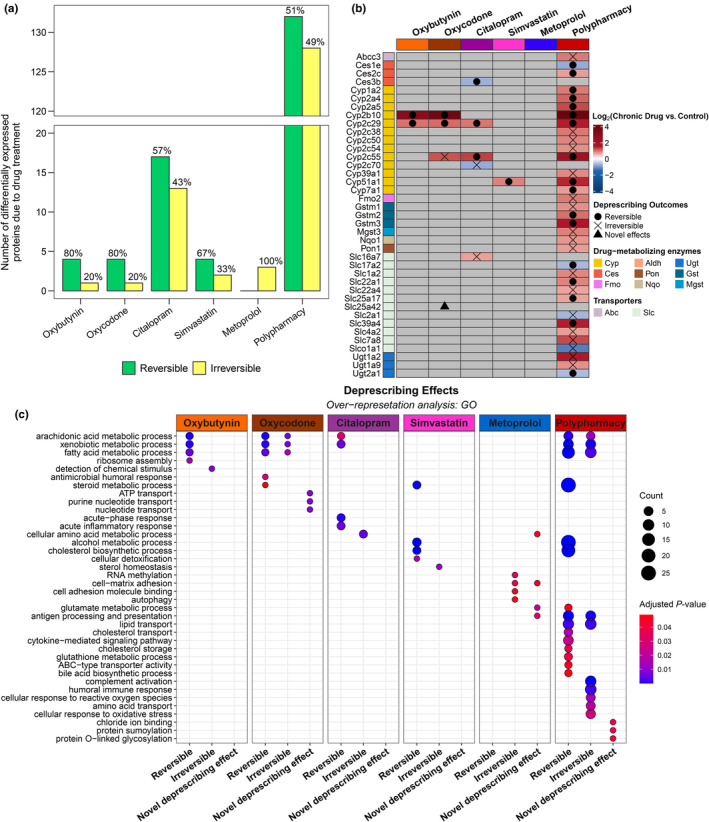
Different molecular effects of deprescribing medications. (a) Grouped bar plot illustrates the proportion of differentially expressed proteins (determined from the comparison of chronic drug versus control) that were reversible or irreversible upon deprescribing (based on chronic drug versus deprescribing; see Figure S7 for different deprescribing outcomes and Table S2 for differential expression analysis results). Raw protein count recorded on the *y*‐axis and percentage (%) was noted in the figure. (b) Heatmap summary of the different deprescribing outcomes on drug‐metabolizing enzymes and transporters (see full data on Figure S8 and Table S2). Chronic drug effects (against control) were color‐coded based on log_2_(fold change [FC]) (red indicating upregulation and blue indicating downregulation). Only significant results were colored accordingly (false discovery rate [FDR]‐adjusted *p* < 0.10 and FC > ±1.50) and non‐significant comparisons were colored gray. Shapes indicate deprescribing outcomes: (i) reversible (circle), (ii) irreversible (cross), and novel deprescribing effect (triangle). Drug‐metabolizing enzyme and transporter classes were annotated. (c) Dot plot summarizing pathway analysis result on selected gene ontology (GO) terms for reversible, irreversible, and novel deprescribing effects in each chronic drug treatment. Size of dots represents the protein count of a given term and colors represent FDR‐adjusted *p*‐value.

Monotherapy effects were reversible after deprescribing, except for metoprolol for which all three proteins were irreversible. For the remaining monotherapies, deprescribing protein expression reversibility potential ranges from 57% (citalopram, 17/30) to 80% (oxybutynin and oxycodone; 4/5) (Figures 4a and S7 for technical explanation of deprescribing outcomes; Figure S8 and Table S9 for complete list of deprescribing outcomes). Deprescribing oxybutynin and oxycodone monotherapies reversed the increased expression of drug‐metabolizing enzymes (Figure 4b,c; Table S10), while deprescribing citalopram monotherapy reversed inflammatory proteins and deprescribing simvastatin reversed cholesterol biosynthetic proteins (Figure 4c; Table S10). Upon probing for irreversible effects, expression of Rtp4 (oxybutynin), Cyp2c55 (oxycodone), Anxa13 (simvastatin), and Tmem97 (simvastatin) remained unaltered following deprescribing. After deprescribing citalopram, amino acid metabolic protein expression changes (Gls2, Afmid, and Acmsd) were irreversible. In addition to reversible and irreversible effects, we also discovered significant protein expression changes that were observed exclusively after deprescribing but not in chronic drug treatment (Figure S7e), which we termed novel deprescribing effects. We found that deprescribing oxycodone and metoprolol monotherapies induced alterations of three (Ccdc117, Atp6ap1, and Slc25a42) and four proteins (Cd74, Tat, Ppm1l, and Epdr1), respectively (Figures 4c and S8).

Complete deprescribing of polypharmacy reversed 50.77% proteins (132/260)—more specifically, 50.79% up‐ (96/189) and 50.70% downregulated (36/71) proteins (Figures 4a and S7a–d). Deprescribing polypharmacy reversed the expression of proteins associated with cholesterol biosynthesis and amino acid metabolism, while complement system and transporter protein alterations persisted (Figures 4c and S8; Table S10). Moreover, some drug‐metabolizing enzymes (e.g., Cyp2a4, Cyp2b10, Cyp2c55, Ces2c, and Gstm3) and immune‐related proteins were reversible with deprescribing, whereas others (e.g., Cyp2c54, Gstm1, Aldh1b1, and Ugt2a1) were not (Figures 4c,b and S8). Some proteins categorized as “irreversible” displayed a high degree of inter‐individual variability upon deprescribing (Figure S7d). Furthermore, five proteins (i.e., Amy2, Lztr1, C1galt1c1, Pias1, and Tmem14c) exhibited novel deprescribing effect (Figures S7e and S8). Overall, we observed that complete deprescribing reverses chronic drug effects variably, while potentially introducing novel drug‐specific proteome changes.

### 2.5 Both hepatic histology and transcriptomics results consistently suggest major changes produced by polypharmacy

**FIGURE 5:**
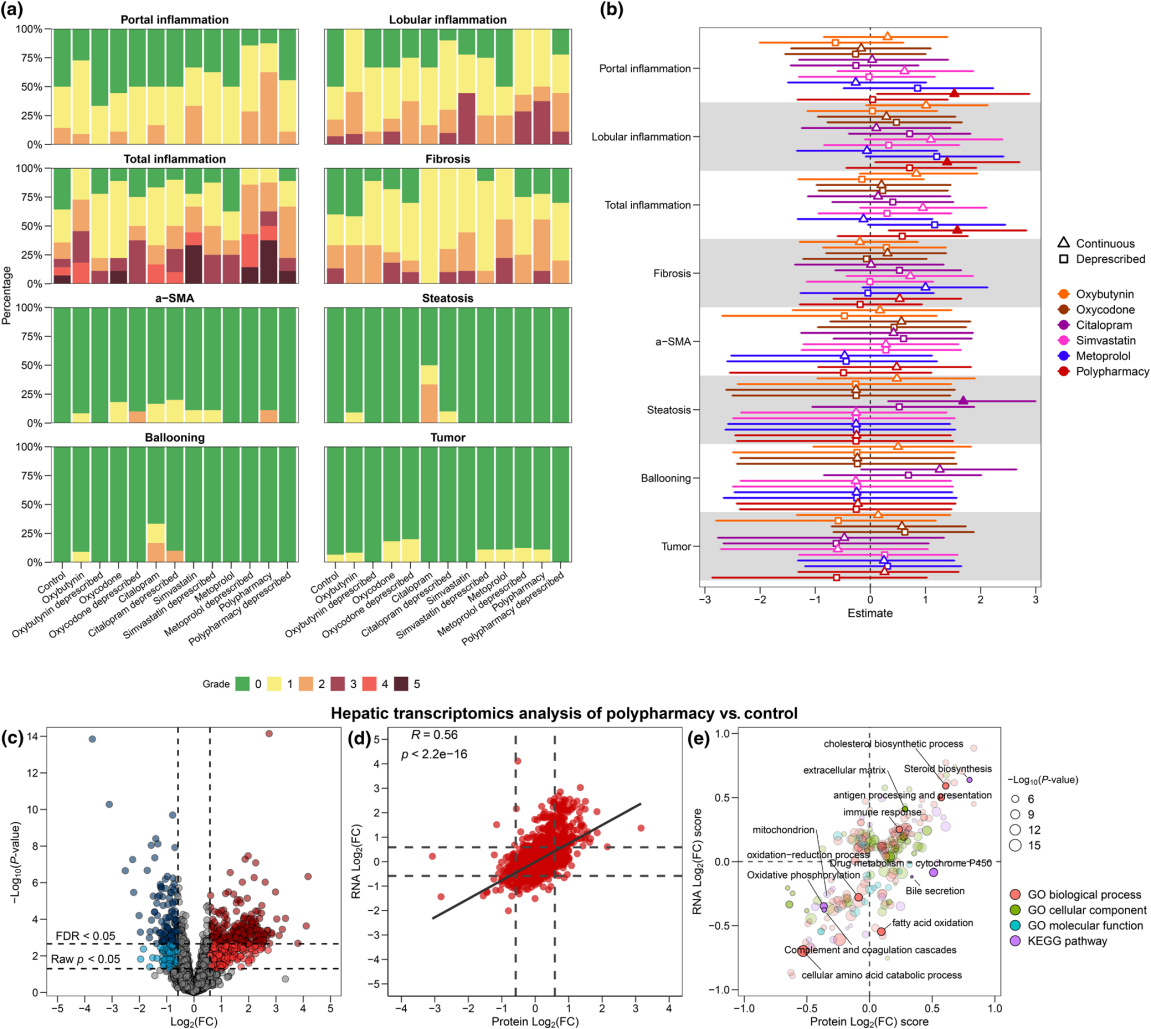
Hepatic histology of chronic monotherapy, polypharmacy, and deprescribing, alongside transcriptome signature of chronic polypharmacy. (a) Stacked bar plots of different histological features across different treatment groups (*n* = 6–15/group). Colors indicate severity of a given histological feature, except for tumors which are binary (0: absent and 1: present). alpha smooth muscle actin (α‐SMA) stains for activated stellate cells. Sample sizes for hepatic histological analysis: Control, *n* = 14–15; oxybutynin, *n* = 11–12; oxycodone, *n* = 9–11; citalopram, *n* = 6, simvastatin, *n* = 6; metoprolol, *n* = 8–9; polypharmacy, *n* = 8–9; oxybutynin deprescribed, *n* = 9; oxycodone deprescribed, *n* = 8–10; citalopram deprescribed, *n* = 10, simvastatin deprescribed, *n* = 8–9; metoprolol deprescribed, *n* = 9–10; polypharmacy deprescribed, *n* = 9–10. (b) Estimates of ordinal logistic regression with Bayes framework, except for tumors, for which logistic regression was applied. Control was used as a reference group. Error bars depict 99% credible interval (CI) and statistical significance is considered when 99% CI does not cross 0 and critical value of *z* > 2.575. Estimates (*x*‐axis) are displayed in log‐scale. Significant estimates are marked with color‐filled mean points. A negative significant estimate (<0) suggests that a given treatment has a lower histological score compared with control whereas a positive significant estimate (>0) suggest a higher one. (c) Volcano plot depicting polypharmacy versus control in the transcriptome level (*n* = 6/group). Vertical lines represent ±1.50 fold change (FC) and the horizontal lines depict raw *p* < 0.05 and false discovery rate (FDR)‐adjusted *p* < 0.05. (d) Scatterplot of RNA (*y*‐axis) and protein (*x*‐axis) log_2_(FC) derived from the differential expression analysis (polypharmacy versus control) on 5967 protein–transcript pairs. Pearson's correlation coefficients (*R*) and *p*‐values are shown. Dotted lines represent ±1.50 FC. (e) Pathway‐level comparison as 2D annotation enrichment plot of log_2_(FC) enrichment score at the RNA (*y*‐axis) and protein (*x*‐axis) levels. Higher positive log_2_(FC) score represents greater degree of upregulation, and vice versa for downregulation. A score at or near 0 indicates that a given pathway experienced no change. The 2D plot can be divided into four quadrants to indicate the concordance and discordance of regulation directionality between the proteome and the transcriptome. Gene ontology (GO) terms and Kyoto encyclopedia of genes and genome (KEGG) pathways are color‐coded, and selected enrichment are highlighted. All enrichment data point depicted are significant enrichment (FDR <0.01) with dot sizes representing *p*‐values.

We next investigated the effect of chronic pharmacotherapy and deprescribing in terms of histological dynamics (*n* = 6–15/group; Figure 5a; Table S1). Applying ordinal logistic regression modelling whereby estimates are log‐scaled, chronic polypharmacy compared with control increased the risk of portal (estimate: 1.53 [99% CI: 0.21, 2.94]), lobular (estimate: 1.37 [99% CI: 0.17, 2.66]), and total inflammation (estimate: 1.56, 99% CI: 0.30, 2.85) (Figure 5b). Changes in inflammation features are consistent with the immune‐related proteome changes observed in polypharmacy. Additionally, chronic citalopram monotherapy leads to higher steatosis grade (estimate: 1.70 [99% CI: 0.43, 3.01]) compared with control (Figure 5b). No significant differences across treatment groups were identified for fibrosis, alpha smooth muscle actin (α‐SMA), ballooning, and tumor burden.

With the prominent effects of polypharmacy on the hepatic proteome and histology, we then sought to explore whether those effects also impact the transcriptome. To do that, we performed bulk RNA sequencing (RNA‐seq) on the livers from control and polypharmacy‐treated mice (*n* = 6/group), from which we had also obtained proteomic data. When we compared polypharmacy‐treated livers to control, we observed similarly large changes—235 upregulated and 136 downregulated transcripts (FDR‐adjusted *p* < 0.05; FC > ±1.50) (Figure 5c; Table S11). To assess the relationship between the proteome and the transcriptome, a total of 5967 protein–transcript pairs were matched. A moderate correlation was observed between the proteome and the transcriptome (*R* = 0.56; *p* < 0.05), demonstrating the directional concordance between proteome and transcriptome effects in response to chronic polypharmacy (Figure 5d). We also assessed the similarities of the proteome and transcriptome at the pathway level and observed that a majority of pathways were affected with the same directionality (Figure 5e; Table S12). Specifically, immune response and cholesterol biosynthesis were upregulated whereas complement system and amino acid metabolism were downregulated at both the protein and transcript levels. Interestingly, the impact on drug‐metabolizing enzymes was solely evident at the proteome level. Altogether, this suggests that most proteome changes in response to polypharmacy were transcriptionally regulated.

RNA‐seq was also performed on polypharmacy‐deprescribed mice (*n* = 3). We observed 131 up‐ and 76 downregulated transcripts when comparing polypharmacy with deprescribed (FDR‐adjusted *p* < 0.05; FC > ±1.50) and no differentially expressed transcripts when comparing deprescribed with control (FDR‐adjusted *p* > 0.05), suggesting a complete reversibility (Figure S9a; Table S11). Deprescribing polypharmacy yielded consistent results in both the transcript/protein level and pathway level (Figure S9b,c; Table S12). Transcriptomic differences between deprescribed polypharmacy and control were minimal, with more effects seen on the proteome (Figure S9c; Table S12).

### 2.6 Hepatic proteome reorganization is associated with clinically relevant phenotypes, including geriatric behavioral outcomes

We were next interested in finding out whether the hepatic proteome alterations are related to clinically relevant phenotypes. Prior investigation documented treatment group differences in several behavioral outcomes, but no clinically relevant differences in serum biochemistry and cytokine levels (Mach, Gemikonakli, et al., 2021; Wu et al., 2022). Here, we applied weighted gene co‐expression network analysis (WGCNA) (Langfelder & Horvath, 2008), a data‐driven analysis that allows unsupervised partitioning of the proteome based on expression patterns across samples into co‐expressed sets of proteins (modules). Using 6489 proteins across all treatment groups (*n* = 75; see methods), WGCNA organized the proteome into 22 modules, labelled as M1 to M22 ordered based on size (M1, *n* = 863; M22, *n* = 74) with M0 designed for unassigned proteins (Figure S10; Table S13). Different modules were enriched with diverse and different pathways (Tables S14 and S15).

**FIGURE 6:**
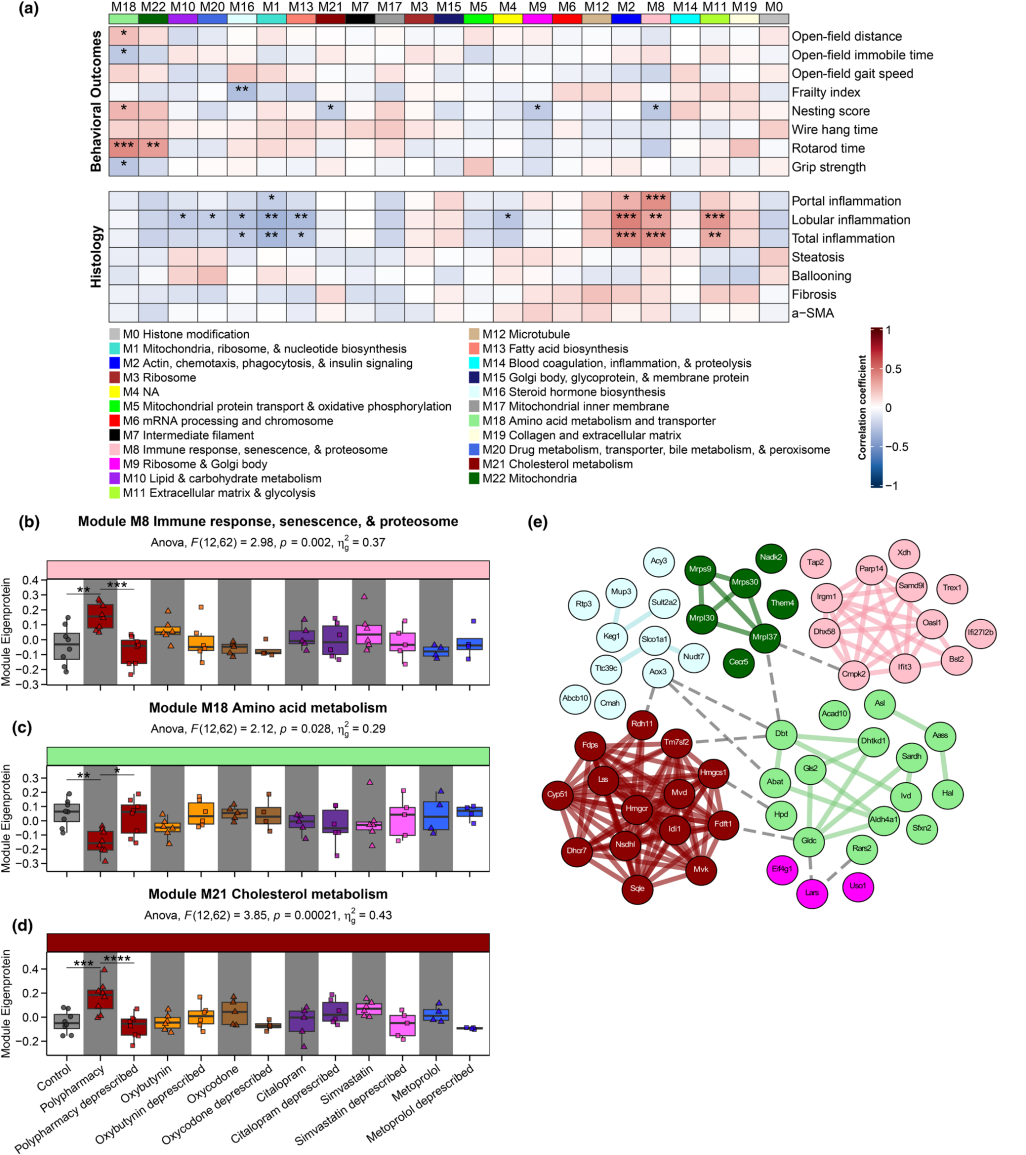
Co‐expression network analysis of the hepatic proteome. (a) Weighted gene co‐expression network analysis (WGCNA) partitioned the proteome into 22 different co‐expressed modules, with module M0 (gray) for unassigned proteins. Heatmap summarizing module–trait relationship analysis between protein modules (columns) and different clinically relevant phenotypes (rows). Only behavioral outcomes and hepatic histology are displayed here and the other traits are in Figure S12. Degree of positive (red) and negative (blue) correlation are shown, with asterisks representing significance (*p < 0.05, **p < 0.01, ***p < 0.001) based on biweight midcorrelation analysis for behavioral outcomes and Kendall's tau correlation analysis for histology. Based on gene ontology (GO) and Kyoto encyclopedia of genes and genome (KEGG) enrichment analysis, selected functional categories of each module were noted at the bottom of the heatmap with their corresponding color codes displayed at the top of the heatmap. Modules of interest were modules that displayed association(s) with any of the geriatric behavioral outcomes, which include (b) M8, (c) M18, and (d) M21. One‐way ANOVA statistics, including degrees of freedom, *F* statistics, *p*‐value, and effect size (*η* ^2^) were recorded. Tukey's post hoc comparisons were denoted as asterisks: *p < 0.05, **p < 0.01, ***p < 0.001, and ****p < 0.0001. (e) Search Tool for the Retrieval of Interacting Genes/Proteins (STRING) analysis generated a protein–protein interaction network of 62 hub protein nodes with 137 edges (average number of neighbors = 6.90; clustering coefficient = 0.65). Hub proteins were identified from the six geriatric outcome‐related modules: (i) M8 immune response, senescence, and proteosome (*n* = 12), (ii) M9 ribosome and Golgi body (*n* = 3), (iii) M16 steroid hormone biosynthesis (*n* = 11), (iv) M18 amino acid metabolism and transporters (*n* = 15), (v) M21 cholesterol metabolism (*n* = 14), and (vi) M22 mitochondria (*n* = 7). Node color coding is based on the designated module colors. Intra‐modular (within‐module) interactions were denoted with thicker lines and color‐coded with the corresponding module colors, whereas inter‐modular (between‐module) interactions were noted as thin dotted gray edges.

The module eigenprotein values for each sample, calculated as the first principal component of the module, were correlated with 66 clinically relevant phenotypic traits, which can be divided into five trait categories: (i) functional/behavioral outcomes, (ii) hepatic histology, (iii) weights, (iv) serum biochemistry, and (v) serum cytokine levels. This higher‐order analysis allows a systems‐level mechanistic appreciation of the relationship between protein dynamics and phenotype that would have otherwise been missed by protein‐level analysis alone. Overall, 129 positive and 102 negative significant correlations were observed (*p* < 0.05) across all trait categories (Figures 6a and S11). Immune‐related modules (M2 and M8) were positively correlated with inflammation histology results. We next focused on protein modules correlated with the geriatric behavioral outcomes, which include modules M18 (amino acid metabolism), M22 (mitochondria), M16 (steroid hormone biosynthesis), M21 (cholesterol metabolism), M9 (ribosome and Golgi body), and M8 (immune response, senescence, and proteasome). Module M18 had the highest number of significant correlations with behavioral outcomes, which includes positive correlations with open‐field distance (*R* = 0.23), rotarod time (*R* = 0.41), nesting score (a measure of activities of daily living) (*R* = 0.26), as well as negative correlations with open‐field immobile time (*R* = −0.25) and grip strength (*R* = −0.26). This suggests a possible association between hepatic amino acid metabolic proteins with motor performance and physical function. Nesting score was also correlated with modules M21 (*R* = −0.22), M9 (*R* = −0.24), and M8 (*R* = −0.25). Module M22 was positively correlated with rotarod time (*R* = 0.34). A negative correlation was observed between the frailty index and module M16 (*R* = −0.30). When comparing the module eigenprotein across treatment groups, M8, M18, and M21 were significantly different in polypharmacy compared with control, and reversed with deprescribing (*p* < 0.05) (Figures 6b–d and S12a–c). Subsequently, we endeavored to identify the potentially important proteins within the modules of interest, known as hub proteins. These proteins have disproportionally high connectivity within the co‐expression network and may be the primary driver of a biological response. We identified a total of 62 hub proteins across the six modules (Figure 6e). The expression profile of these hub proteins across the samples created a cluster for chronic polypharmacy, distinct from the majority of control, chronic monotherapy, and deprescribing samples (Figure S12d), suggesting a potential role for these hepatic proteins in polypharmacy‐related adverse geriatric outcomes.

## 3 DISCUSSION

We present a comprehensive survey of the murine hepatic proteome dynamics following chronic monotherapy and polypharmacy from middle age to old age, and deprescribing in old age. Individual monotherapy effects were minor, exhibiting either drug‐specific or non‐specific effects. Major proteome perturbation was only apparent in polypharmacy, through primarily sub‐additive regulation. Adaptive pharmacokinetics responses to polypharmacy through hepatic protein expression dynamics occurred, whereby higher serum drug and metabolite levels were observed in polypharmacy than the constituent monotherapies. Other major polypharmacy effects include proteins involved in immune‐related and metabolic pathways. Complete deprescribing reversed some drug effects; however, we also observed the introduction of novel hepatic proteome changes. The drug‐induced hepatic changes were associated with clinical and behavioral geriatric outcomes.

Though most of the proteins were sub‐additively regulated and monotherapy effects were reflected in the polypharmacy signature, we demonstrated that a greater extent of change was induced by polypharmacy compared with monotherapy. The liver adaptively responded to chronic exposure to polypharmacy by altering the expression of DMETs, which led to an increase in serum drug metabolite levels. Though none of the medications are typically regarded as CYP inducers (Xiong et al., 2022)—rather citalopram exhibits weak CYP inhibition (Jeppesen et al., 1996; Spina et al., 2008), we observed that oxycodone and oxybutynin, in chronic monotherapy and polypharmacy, increased CYP expression. Induction of Cyp2b10 has been previously observed following exposures to chronic binge drinking, phenytoin, pentobarbital, and fasting (de Vries et al., 2016; Jackson et al., 2004; Mackowiak et al., 2022). The transcriptomic evidence demonstrated that polypharmacy‐associated changes in CYP expression were not mediated through transcriptional regulation. This suggests that the changes observed emerge from other mechanisms, such as post‐translational modifications and/or protein degradation rates (Aguiar et al., 2005; de Jong et al., 2020).

Despite the increase in DMET expression, serum levels of parent drug species were also higher with polypharmacy than the corresponding monotherapies, though metabolite‐to‐parent ratio remained unaltered. This suggests that polypharmacy leads to an overall higher drug exposure compared with monotherapy even though dose administered is the same. Differences in serum parent drug or metabolite levels were drug‐specific, suggesting a difference in drug metabolism prioritization which may depend on enzyme affinity, drug distribution, dosing regimen, availability of alternative metabolic pathways, and/or differences in elimination rates, among others (Herrlinger & Klotz, 2001). Future pharmacokinetic modelling could shed further light on the mechanisms. Nevertheless, with polypharmacy, higher serum drug levels along with exposure to multiple medications with different mechanisms of action may consequently lead to greater proteome perturbations in the liver and potentially other organs.

The altered expression of DMETs may introduce unintended molecular consequences. Ongoing drug metabolism requires energy and cofactors (Cordes et al., 2018), potentially diverting resources from other hepatic processes and compromising homeostatic metabolic networks. Besides drug metabolism, CYP enzymes also participate in various lipid metabolic processes (Shoieb et al., 2020; Silvia et al., 2006), further contributing to drug‐induced metabolic perturbations. These metabolic changes along with increased drug exposure may contribute to the observed polypharmacy‐induced dysregulation such as immune‐related, cholesterol metabolic, and amino acid metabolic proteins, among others, and contribute to adverse geriatric outcomes. These changes were also consistent in the transcriptome. Moreover, such immune‐related changes were further demonstrated in hepatic histology results. Immune function, a recognized hallmark of aging (Franceschi et al., 2018), has been documented to be modulated by different medications. Medications with anticholinergic properties (e.g., oxybutynin, oxycodone, and citalopram) are linked to inflammation in serum (Sanghavi et al., 2022) and brain (Yoshiyama et al., 2012). Both simvastatin (Arnaud et al., 2005; Jain & Ridker, 2005) and metoprolol (Clemente‐Moragón et al., 2020; Clemente‐Moragón et al., 2021) have been shown to exhibit anti‐inflammatory effects. Notably, marked increased expression of major histocompatability complex (MHC) subunits was observed with the use of polypharmacy, and similar changes have been characterized with aging liver (Almanzar et al., 2020). Furthermore, inflammation is known to affect CYP‐mediated drug metabolism (de Jong et al., 2020; Stanke‐Labesque et al., 2020; Wang et al., 2022), potentially influencing serum drug levels. Polypharmacy also influenced the expression of complement system effectors, which may potentially affect aging and age‐related diseases (Zheng et al., 2022). Concurrently, CYP enzymes also may elicit hepatoprotective effects; for instance, Cyp2b10 has been shown to protect against steatosis and fibrosis (Mackowiak et al., 2022). Dynamics of cholesterol biosynthetic enzymes in polypharmacy may be predominantly driven by simvastatin, some of which are caused by compensatory mechanisms (Reihnér et al., 1990), which may have implications in discontinuation (Thomas et al., 2023). In addition to immune response, polypharmacy‐induced dysregulation of metabolic and proteolytic enzymes also aligns with the key hallmarks of aging—that is, disrupted nutrient signaling and protein homeostasis, respectively (López‐Otín et al., 2023). Overall, changes in DMET expression, especially in polypharmacy, may produce pleiotropic effects, leading to pharmacokinetic, pharmacodynamic, and aging‐related consequences. Further investigation into longitudinal studies with different chronic administration periods, CYP activity assays, and metabolomics profiling may elucidate these complex interactions.

To our knowledge, this study is the first to characterize the molecular profile of deprescribing polypharmacy and we identified several key findings. First, major changes in the hepatic proteome with chronic polypharmacy support gradual dose reduction (tapering) to facilitate the time needed for the homeostatic adjustment, and the need for close monitoring of deprescribing. Second, deprescribing can reverse many drug‐induced molecular changes in the healthy‐aged liver. When measured several months after complete deprescribing, the prominent drug effects were reversed and the majority of “irreversible” proteins have high inter‐individual variability upon deprescribing. Third, we identified some novel deprescribing effects from withdrawing oxycodone, metoprolol, and polypharmacy. One of the novel deprescribing oxycodone effects is the altered expression of Ccdc117, which has been linked with opioid use (Falconnier et al., 2023). Moreover, novel changes in Tat and Epdr1 expression were observed following deprescribing of metoprolol, which may be caused by changes in adrenergic signaling (Andersson, 1983; Deshmukh et al., 2019). With polypharmacy, novel deprescribing changes of Pias1 may have implications for inflammatory response (Luo et al., 2022). Beyond informing effectiveness of deprescribing through the potential of molecular reversibility, the molecular characterization of deprescribing, with a focus on novel effects in conjunction with irreversible effects, may elucidate mechanisms of medication withdrawal/discontinuation syndromes and consequently inform deprescribing guidelines. Indeed, currently it is a challenge to determine whether the novel and irreversible molecular effects are beneficial or adverse effects and more work is needed. Future studies of deprescribing should explore different deprescribing implementation designs with considerations in (i) number of medications deprescribed, (ii) complete halt versus dose reduction, (iii) abrupt versus gradual cessation, (iv) concurrent versus stepwise deprescribing of multiple medications, and/or (v) investigation of changes over time throughout the deprescribing process.

Furthermore, our study identified several hepatic protein classes that are associated with important clinically relevant outcomes, such as mobility, frailty, and activities of daily living. The association between hepatic amino acid metabolic proteins and geriatric behavioral outcomes was the most striking, whereby the reduction in hepatic amino acid metabolic proteins as observed in polypharmacy may influence systemic exposure to amino acids and plasma nitrogen imbalance. Given the reported association between metabolic dysregulation and sarcopenia (Lu et al., 2020; Okun et al., 2021; Qiu et al., 2012), a possible mechanism of drug‐related physical impairment may be mediated through liver‐muscle crosstalk. For example, one of the hub proteins of this module is Gls2 which is involved in glutamine catabolism and contributes to age‐related changes (Adachi et al., 2018; Dhahbi et al., 1999; Meynial‐Denis, 2016). We also observed a negative correlation between the clinical frailty index and hepatic steroid hormone biosynthesis. The liver plays an important role in steroid hormone regulation through cholesterol biosynthesis (Charni‐Natan et al., 2019). Consistently, lower serum cholesterol levels and higher remnant cholesterol are associated with frailty or frailty risk (Hu et al., 2023). In addition, serum testosterone declines with age in men and contributes to skeletal muscle mass, suggesting that steroid hormonal imbalance also may contribute to adverse geriatric outcomes (Eichholzer et al., 2012). The negative association between activities of daily living and immune‐related and cholesterol metabolic proteins in the liver is indirect, even in our preclinical model, without the socio‐demographic factors that contribute to older adults. Interestingly, a neuroimmunoendocrine (França et al., 2023; Hammami et al., 2020) and microbiome (Gemikonakli et al., 2023; Shimizu, 2018) link with activities of daily living has been proposed. Overall, the low‐to‐moderate correlations of the hepatic proteome with geriatric behavioral outcomes may suggest an indirect relationship between the two, presumably mediated by processes occurring in other organs such as the brain and skeletal muscles.

## 4 CONCLUSION

Our study advances the understanding of the underlying mechanisms by which polypharmacy and deprescribing may affect hepatic and global function in old age. Through the aged murine model, we extensively characterized the patterns of chronic monotherapy and polypharmacy drug‐induced hepatic proteome rearrangement, whereby polypharmacy caused changes not seen with chronic monotherapies. We also showed polypharmacy changes are complex and include upregulating immune‐related, cholesterol‐metabolic, and drug‐metabolizing proteins, and elevating serum drug/metabolite levels. Importantly, deprescribing has reversible, irreversible, and novel proteome effects, which need further investigation to understand their clinical impact. Finally, our findings with hub proteins related to functional outcomes suggest cellular and molecular changes that potentially contribute to adverse geriatric outcomes. Ultimately, this study lays the foundational framework that we hope will guide future research into the mechanisms of polypharmacy and deprescribing to inform optimal medication use for older adults.

## 5 METHODS

### 5.1 Animals

Healthy male C57BL/6J (B6) mice were sourced and housed from the Kearns facility, Kolling Institute, Sydney, Australia. All experimental procedures were approved by the Northern Sydney Local Health District Animal Ethics Committee, Sydney, Australia. Husbandry details are described previously (Mach, Gemikonakli, et al., 2021). Briefly, animals were checked twice weekly to ensure their well‐being and ethical use of animals in research. They were assessed via macroscopic and phenotypic assessments in vivo, assisted by a veterinarian.

### 5.2 Pharmacological treatment and deprescribing

The treatment regimen and deprescribing procedure are described in Mach, Gemikonakli, et al. (2021). Briefly, animals were administered medication in their feeding chow (oxybutynin, citalopram, simvastatin, and metoprolol) or water (oxycodone). Animals began treatment at 12 months to 26 months. At 21 months of age, animals were re‐randomized to continue or deprescribed treatment via stratification of the two groups to have to have similar clinical frailty index scores at 21 months.

The doses were the therapeutic oral dose derived from previous studies of administering these medications in monotherapy in mice. This dose was used to calculate the medication required to mix into control diet by Specialty Feeds, based on observed food intake of 0.11 g food/g mouse/day. Oxycodone was administered in drinking water to comply with scheduled drug regulations. Food and water intake (g of food or mL of water/g of mouse/day) were determined as an average of 2 weeks prior to behavioral testing. Animals in the same cage received an average for food and water intake.

### 5.3 Behavioral assessments

Functional behavioral assays were performed, as previously described (Mach, Gemikonakli, et al., 2021), and only the 24‐month data were relevant to this study, as the closest time point to termination. These tests assessed mobility (open‐field distance, immobile time, and gait speed), frailty (Mouse Clinical Frailty Index), activities of daily living (nesting score), balance and motor coordination (wire hang time and rotarod time), and forelimb grip strength.

### 5.4 Tissue collection

Serum was collected at age 24 months from submandibular vein and spun down with 1.1 mL serum Z‐Gel microtube (41.1378.005, SARSTEDT, Australia). The portal vein was then cannulated with an 23G catheter (BD, Sydney, Australia) and the liver was perfused with oxygenated Krebs‐Henseleit Bicarbonate buffer (95% O_2_, 5% CO_2_, 37^o^C) to remove blood from the liver tissue. Following all behavioral experiments, animals were euthanized at 26 months of age. Body and liver weight were recorded, and liver tissue was collected and snap‐frozen in liquid nitrogen. Samples were stored in a −80°C freezer before processing. This study is a part of a larger study that started with 340 animals, of which 116 were analyzed for histology. Mice that were found dead or had to be euthanized due to illness or disease before the tissue harvest point of 26 months of age (10.8%, 37/340 from the original colony) or those from pre‐defined cohorts used for longevity analysis (i.e., survival monitored beyond age 26 months) were excluded from the analysis. Of the 116 animals with tissue collected at aged 26 months, a subset were analyzed for proteomics (*n* = 79) and transcriptomics (*n* = 15). Proteomics samples were randomly selected from each treatment group based on the availability of in vivo functional data. Having hepatic tumors was not an exclusion criterion. We observed a 3.8% prevalence of hepatic tumors in proteomics animals (3/79): one polypharmacy, one oxycodone deprescribed, and one simvastatin deprescribed.

### 5.5 Serum biochemistry

Serum electrolytes, liver function tests, and creatinine were analyzed by a National Association of Testing Authorities‐accredited hospital laboratory, PaLMS (Pacific Laboratory Medicine Services) at Royal North Shore Hospital (Sydney, Australia), using an Abbott Architect Clinical Chemistry Analyzer.

### 5.6 Serum drug levels

Serum drug levels were measured following the validated protocol previously described (Mach, Wang, & Hilmer, 2021). Differences in mean was compared using a two‐tailed Student's *t* test and *p* < 0.05 was considered significant.

### 5.7 Serum cytokine levels

Serum cytokine concentrations were and prepared and measured using a multiplex immunoassay at the Australian Proteome Analysis Facility as described previously (Wu et al., 2022).

### 5.8 Proteomics

Proteins were extracted from homogenized whole liver tissue samples consisting of all the lobes, excluding the median lobe that was used for histology. After cryogenic grinding, powdered liver samples were lysed by incubation and homogenization in 5.0% SDS, 50 mM TEAB (pH 8.5). Samples were treated with benzonase and clarified by centrifugation (15,000 g for 2 min 30 s) to obtain soluble proteins. Next, 46 μL aliquot of the supernatant (~100 μg proteins) were reduced with 5 mM TCEP for 15 min at 55°C, alkylated with 20 mM iodoacetamide for 10 min incubation at room temperature, and acidified with 2.5% aqueous phosphoric acid. Binding/wash buffer (100 mM TEAB in 90% methanol) was added to the protein supernatant prior to transferring into the S‐trap column (Zougman et al., 2014) (Protifi, USA). S‐traps with bound proteins were washed repeatedly with the binding/wash buffer to remove residues. MS‐grade trypsin (Promega), dissolved in 50 mM TEAB, was applied to the column and digestion occurred overnight at 37°C. Peptides were eluted from the column using 50 mM TEAB followed by 0.2% formic acid and subsequently 50% acetonitrile. Pooled peptides were dried using a SpeedVac system (Thermo Fisher Scientific) at 35°C and then resuspended with 0.1% formic acid. Peptide concentration was normalized to 1.0 μg/μL as determined with the NanoDrop spectrophotometer (Thermo Fisher Scientific).

Peptides digests were quantified in a randomized batch by liquid chromatography‐tandem mass spectrometry (LC–MS/MS) operating in data‐independent acquisition mode using a nano‐ultra‐pressure‐LC system (Ultimate 3000 RSLCnano; Thermo Fisher Scientific) coupled to an orbitrap MS (QExactive HF‐X; Thermo Fisher Scientific) as described previously (Steffen et al., 2021).

Protein identification and label‐free quantification were performed using DIA‐NN (v1.8) (Demichev et al., 2020). Using library‐free mode, either .*mzML* files following demultiplexing with ProteoWizard (Kessner et al., 2008) or .*raw* files were searched against the *Mus musculus* proteome FASTA library (containing 17,014 protein entries) retrieved from UniProt in August 2019. The *match‐with‐run* function was used with the following settings: (i) maximum of two missed tryptic cleavages and (ii) three allowed variable modifications (*N*‐term M excision, carbamidomethylation, and methionine oxidation). Peptides considered were (i) between 7 and 30 amino acids with (ii) precursor charge between 1 and 4, (iii) mass range of 300–1800 *m*/*z*, and (iv) product ion mass range of 200–1800 *m/z*. Peptide and protein identification FDR confidence was set to 1%.

### 5.9 Bioinformatics analysis

Data analysis and visualization were performed primarily in R/Rstudio (v4.2.0) (R Core Team, 2022) using ggplot2 (v3.4.2) (Wickham, 2016). We initially quantified 10,577 unique proteins. Proteins with missing values in 60% of samples per treatment group were filtered out, leaving 6489 proteins for analysis. Data acquisition occurred across two MS runs and after log_2_‐transformation and quantile normalization, ComBat‐based batch effect correction was performed using proBatch (v.1.11.0) (Čuklina et al., 2021). Following that, six outliers were identified using robust PCA available in rrcov (v1.7–4) (Chen et al., 2020; Todorov & Filzmoser, 2009) and subsequently removed. Finally, NAGuideR (v0.2.0) (Wang et al., 2020) was used to select the most optimal missing values imputation strategy across 23 different methods. Accordingly, imputation was conducted with robust sequential imputation (impseqrob) (Branden & Verboven, 2009), which was determined to be the most appropriate imputation algorithm based on classical criteria.

PCA was computed using *prcomp()* function in R. Differential expression analysis was conducted through a robust linear model with empirical Bayes moderation using the limma (v3.54.2) (Ritchie et al., 2015) package. Birth cohort was included as a covariate. Differentially expressed protein was defined as a protein with FDR‐adjusted *p* < 0.10 and > ±1.50 FC (i.e., >1.50 or <0.67). Using ComplexHeatmap (v2.14.0) (Gu et al., 2016), heatmap of differentially expressed proteins across the monotherapy and polypharmacy signatures was generated after row *z*‐score scaling and semi‐supervised hierarchical clustering analysis (HCA). Specifically, treatment allocation of each sample was first identified and clustered together, and column HCA was applied between‐treatment and within‐treatment separately. Unsupervised clustering was applied for row HCA. Whenever appropriate, unsupervised HCA was performed with Euclidean distance and complete linkage. To visualizes the differentially expressed protein overlaps between different groups, ComplexUpset was used (v1.3.3) (Lex et al., 2014). Pathway analyses (over‐representation analysis [ORA] and GSEA) were performed with clusterProfiler (v4.6.2) (Wu et al., 2021) and org.Mm.eg.db (v3.16.0) on gene ontology (GO) terms. An enriched pathway was considered as statistically significant when FDR‐adjusted *p* < 0.05 by the Benjamini–Hochberg method.

To explore synergism, we modified the drug response additivity model formulated by Megger et al. (2018) to accommodate our polypharmacy regimen, as the following:

$$\text{Synergistic score}=\frac{\log_{2}\left(P\right)}{\log_{2}\left(m_{1}\right)+\log_{2}\left(m_{2}\right)+\log_{2}\left(m_{3}\right)+\log_{2}\left(m_{4}\right)+\log_{2}\left(m_{5}\right)}$$

where *P* is the FC of polypharmacy against control, and *m*_n_ is the FC of monotherapy *n* against control. Using the formula above, the synergistic score was dichotomized to >1.00 for synergistic effect and ≤1.00 for sub‐additive effect.

The drug that may potentially drive the net change observed within the polypharmacy signature was identified as the monotherapy with the minimum absolute difference with polypharmacy in terms of log_2_(FC). This creates five drug clusters to which ORA was applied. We also conducted a similar analysis in which only sub‐additive polypharmacy effects were considered.

To identify established protein–drug interactions (PDIs), drugs utilized in the polypharmacy regimen were queried on the STITCH database (v5.0) (Szklarczyk et al., 2016). The search was conducted with default settings, except for organism: *Mus musculus* and minimum required interaction score: 0.50, yielding an initial PDI network with 361 protein nodes and 3941 edges. Because tissue‐specific interactions in mice are unavailable in the current STITCH version, the basal murine hepatic gene expression of the STITCH proteins was queried in the Mouse Genome Informatics (MGI) Gene eXpression Database (GXD) (v6.22) (Baldarelli et al., 2021) with assay type: RNA‐seq, sex: male, strain: C57BL/6J, and structure: liver. Expression levels were categorized based on default transcript per million (TPM) cutoffs. Out of the 361 proteins in the PDI network, 11 protein entries were not identified in MGI GXD, leaving 350 proteins for basal hepatic expression analysis. PDI network was visualized in Cytoscape (v3.9.1) (Shannon et al., 2003). To further probe for DMETs, we conducted a manual search for specific protein features. The differential expression analysis outcome of proteins identified in the STITCH network and manual search analysis were then summarized as a heatmap with HCA.

Reversibility potential of deprescribing was determined based on two pairwise comparisons: (i) chronic drug treatment versus control and (ii) chronic drug treatment versus the corresponding deprescribing group. A reversible protein is defined when a given protein is differentially expressed in both comparisons with the same directionality. An irreversible protein is defined when it is differentially expressed comparing chronic drug treatment versus control, but not in chronic drug treatment versus deprescribing. Novel deprescribing effects are identified with three different criteria: (i) not differentially expressed with chronic drug treatment versus control, but is differentially expressed when comparing chronic drug treatment versus deprescribing, or (ii) differentially expressed in both comparisons but at different directionalities, and (iii) differentially expressed between deprescribing and control.

To describe the relationship between the hepatic proteome and clinically relevant phenotypes, a systems approach was applied to generate and analyze co‐expression network of the proteomics dataset using WGCNA (v1.71) (Langfelder & Horvath, 2008). All 6489 proteins were used in WGCNA analysis. We first construct a signed hybrid co‐expression network and partition the proteome into co‐expressed modules, followed by module–trait correlation analysis and hub protein identification.

To construct a co‐expression network and identify co‐expressed protein modules, protein expression data was used to create a pairwise correlation matrix by computing biweight midcorrelation after outlier removal. The correlation matrix was then converted to a weighted adjacency matrix to represent a scale‐free topology where the soft‐threshold power β of 5 (*R* ^2^ > 0.85). A topological overlap matrix (TOM) was generated from the weighted adjacency matrix to include similarity of co‐expression relationship. TOM‐based dissimilarity matrix (1—TOM) was applied to hierarchical clustered protein modules by dynamic tree‐cutting algorithms (deepSplit = 3, minModuleSize = 50, and mergeCutHeight = 0.18).

Module–trait correlation analysis was conducted with biweight midcorrelation, except for histology which used Kendall's tau correlation analysis. A significant correlation was defined as *p* < 0.05. ORA‐based GO and Kyoto encyclopedia of genes and genomes (KEGG) pathway analysis was applied on each module to identify the enriched pathways. MEs were compared across treatment groups wherein ANOVA was performed followed by Tukey post hoc comparisons on drug effects (chronic treatment versus control) and deprescribing effects (chronic treatment versus deprescribing).

Module membership (MM) and gene significance (GS) cutoff parameters were used to define hub proteins of modules associated with behavioral outcomes. MM considers the correlation between individual proteins with the ME values while GS is the protein‐level correlation with the interrogated phenotype. We define hub proteins from a given module with having >0.75 MM and >±0.20. For module 18 which was correlated with multiple behavioral outcomes, proteins were considered hub if their GS was >±0.20 with at least one of these outcomes. In Cytoscape, hub proteins were visualized as a protein‐protein interaction network which was derived from Search Tool for the Retrieval of Interacting Genes/Proteins (STRING) (v12.0) with default settings, except species: *Mus musculus* and minimum required confidence score: 0.50. The expression of the hub proteins across all samples was visualized with a heatmap with unsupervised HCA.

### 5.10 Histology

A subsection of the median hepatic lobe was fixed in 10.0% neutral‐buffered formalin for 24 h and then processed for embedding using a Shandon Excelsior tissue processor (A78400006; Thermo Fisher, England). Paraffin‐embedded livers were sectioned into 5 μm slides. Each liver specimen was stained with hematoxylin and eosin (H&E), Sirius Red, and α‐SMA immunohistochemistry stain for histopathological analysis, general cellular morphology, fibrosis (Batts & Ludwig, 1995), and visual activated hepatic stellate cells, respectively. Staining protocols used were from Solon‐Biet et al. (2014) and Warren et al. (2011). H&E‐stained histological slices were scored for portal (Emad et al., 2010) and lobular inflammation (Jung et al., 2016), steatosis (Jung et al., 2016), ballooning degeneration (Jung et al., 2016), and the presence of tumors. A graded scoring system was applied whereby a student was trained by C.M. (anatomical pathologist) at Royal Price Alfred Hospital, Sydney, Australia, and blinded from the treatment groups throughout the experiment. Specimens suspected of having tumors, very large inflammatory nodules, and/or great proportion of foreign cell types, were excluded upon confirmation by C.M. To compare histological features of the liver, an ordinal logistic regression with *cumulative(probit)* response distribution was fitted on a Bayes framework using *brms* (v2.20.4) R package (Bürkner, 2017), except for tumors which used logistic regression with *bernoulli(probit)* response distribution. Control was used as a reference category, *normal(0, 5)* was used as priors for intercepts and population‐level effects, and 99% credible interval and critical value of *z* > 2.575 were used to determine statistical significance.

### 5.11 Transcriptomics

Transcriptomics analysis was performed only on control, polypharmacy, and the corresponding deprescribing group (*n* = 6/group). TRIzol‐based RNA extraction was conducted on the liver samples, and subsequent RNA work was performed as previously described (MacArthur et al., 2022). RNA quality was quantified using NanoDrop spectrophotometer and Agilent 2100 Bioanalyzer. Illumina TruSeq Stranded Total RNA Sample Preparation and Illumina NovaSeq 6000 with 20 million paired‐end reads (150‐bp length) per sample were used to prepare RNA library and sequencing, respectively. Read alignment and annotation against the GRCm38.p6 mouse genome assembly were performed using Rsubread (v2.3.7) (Liao et al., 2019). We identified one deprescribing sample as an outlier and two failed RNA sample quality control, resulting in *n* = 3 for polypharmacy deprescribed group.

Lowly expressed genes that did not have at least five counts per million in at least five samples were filtered out, yielding 10,584 transcripts from the initial 42,208 transcripts quantified. We next normalized the transcript dataset using relative log expression approach followed by negative binomial generalized linear modelling implemented by DESeq2 (v1.38.1) (Love et al., 2014). Differentially expressed transcripts were defined as FDR‐adjusted *p* < 0.05 and > ±1.50 FC. Omics integration was conducted at the protein/transcript level and the pathway level. Protein–transcript pairs were matched using a common feature identifier. Across the three pairwise comparisons, Pearson's correlation analysis was conducted on log_2_(FC) of the 5967 protein–transcript pairs. Pathway‐level comparison was conducted with 2D annotation enrichment analysis (Cox & Mann, 2012) available in Perseus (v2.0.6.0) (Tyanova et al., 2016) with 1% FDR threshold calculated through Benjamini–Hochberg procedure. Log_2_(FC) was used as the metric and statistical analysis was conducted with GO terms and KEGG pathways.

## AUTHOR CONTRIBUTIONS

S.N.H and D.G.L.C. conceptualized and conceived the project. S.N.H., J.M., and D.G.L.C. designed the study. J.M. coordinated and performed some of the mouse work, including behavioral data acquisition, sample collection, and serum data acquisition. With technical support from M.J.M., K.W. performed the proteomics sample preparation. M.J.M. and M.P.M conducted the proteomics data acquisition, whereas S.J.M. and M.R.M. conducted the transcriptomics data acquisition. K.W. designed the analysis framework and performed all associated analyses with direct inputs from J.M. and S.N.H. M.R.M., S.J.M., M.J.M., and M.P.M. provided the statistical and bioinformatics support. C.M. supervised the histology analysis. K.W. and J.M. wrote the original draft of the paper with oversight and direction from S.N.H. All authors provided the critical feedback and/or edited the manuscript and approved the final version for submission.

## FUNDING INFORMATION

No funding information provided.

## CONFLICT OF INTEREST STATEMENT

No conflict of interest.

## Supporting information


Tables S1–S9.

**Figure S1.** Proteomics preprocessing strategies. (a) Number of proteins quantified in each sample (peptide and protein false discovery rate [FDR] <1%). Mean intensity and boxplot intensity of each sample (b) before and (c) after quantile normalization, color‐coded based on batch. (d) Correlation matrix between samples before and after batch effect correction using ComBat algorithm. (e) Violin plot depicting Pearson’s correlation coefficient between‐batch and within‐batch.

**Figure S2.** Principal component analysis (PCA) and enrichment analysis of chronic monotherapy and polypharmacy. (a) PCA comparing chronic (continuous) drug effects. Samples are color‐coded by drug treatments and shape indicates chronic (triangle) or deprescribed (square) drug treatment. Here, only chronic drug treatment groups and control cluster ellipses (95% confidence) are depicted, with deprescribed samples uncolored. The explained variance (%) is denoted in brackets in the axis titles. (b) Dot plot showing gene set enrichment analysis (GSEA) of selected gene ontology (GO) pathways enriched in chronic drug treatment compared with control, with size of dots proportional to protein ratio and colors representing false discovery rate (FDR)‐adjusted *p*‐value.

**Figure S3.** Identifying dominant drug driver in the polypharmacy regimen with synergism restriction. (a) Similar to Figure 2c, the alluvial plot depicts the potential drug driver of the polypharmacy regimen with the additional filter in which only sub‐additive effects were considered for single drug driver analysis, and the rest are attributed to unique synergistic polypharmacy effect. Protein counts and proportion (%) were shown. (b) Dot plot showing selected gene ontology (GO) pathways enriched in the five “dominant drug” clusters along with the synergistic polypharmacy cluster within the polypharmacy signature, with size of dots proportional to protein counts and colors representing false discovery rate (FDR)‐adjusted *p*‐value.

**Figure S4.** Properties of protein–drug interaction (PDI) network in a murine hepatic system and serum metabolite‐to‐parent ratio. (a) Complete Search tool for Interacting Chemicals (STITCH)‐derived PDI network consisting of 361 protein nodes (circle) and five drug nodes (rhombus) with 3941 edges. Drug nodes and its respective interactions with proteins were color‐coded accordingly. Protein–protein interaction edges were colored as gray. Basal hepatic expression, based on Mouse Genome Informatics Gene eXpression Database (MGI GXD) RNA‐seq data, was annotated with node borders wherein solid line indicates expected expression while dotted line indicates not. Proteins identified in the hepatic proteome dataset were color‐coded as yellow. (b) Barplot of number of direct interactions each drug is known to be involved with. (c) Basal expression of the PDI proteins in C57BL/6J male murine liver. Categories were determined from RNA‐seq data (normalized as transcript per million [TPM]) with default setting: (i) below cutoff (0.00–0.49 TPM), (ii) low (0.50–10.00 TPM), (iii) medium (10.01–1000.00 TPM), and (iv) high (>1000.00 TPM). Proteins that classified as low, medium, or high expression were deemed basally expressed in the liver (bars on the right of the red dotted vertical line). Bars were stacked based on whether the proteins were detected in the proteomics dataset annotated as proportion (%) of total basal hepatic expression per expression categories. (d) Serum levels of drug metabolite‐to‐parent ratios from 24‐month‐old mice comparing polypharmacy against the corresponding monotherapies. Data is represented as mean ± SEM. Two‐tailed Student’s t test was conducted and no significance was identified.

**Figure S5.** Probing for drug‐metabolizing enzymes and transporters. Log_2_(fold change [FC]) heatmap of drug‐metabolizing enzymes (left) and transporters (right) between chronic drug treatment versus control. Heatmap was color‐coded (red indicating upregulation and blue indicating downregulation) and annotated with statistical significance: * false discovery rate (FDR)‐adjusted *p* < 0.10, **p < 0.05, ***p < 0.01, ****p < 0.001. Unsupervised hierarchical clustering was performed with Euclidean distance and complete linkage. Drug‐metabolizing and transporter classes were also annotated. Cyp: cytochrome P450; Ces: carboxylesterase; Fmo: flavin‐containing monooxygenase; Adh: alcohol dehydrogenase; Aldh: aldehyde dehydrogenase; Pon: paraoxonase; Por: P450 oxidoreductase; Nqo: NADPH‐quinone oxidoreductase; Sult: sulfotransferase; Ugt: UDP‐glucuronosyltransferase; Gst: glutathione S‐transferase; Mgst: microsomal glutathione S‐transferases; Nat: N‐acetyltransferase; Comt: catechol‐O‐methyltransferase; Abc: ATP‐binding cassette transporter; Slc: solute carrier transporter.

**Figure S6.** Principal component analysis (PCA) comparing deprescribing treatment groups against control and its corresponding chronic drug treatment groups. Samples are color‐coded with corresponding drug treatments and chronic (triangle; solid 95% confidence ellipses) or deprescribed (square; dotted 95% confidence ellipses) drug treatment. The explained variance (%) is noted in brackets beside the axis titles.

**Figure S7.** Log_2_(fold change [FC]) scatterplots, representative boxplots, and statistical requirements for deprescribing outcomes. (a) Considering chronic drug treatment, its respective deprescribing group, and control, a log_2_(FC) scatterplot of two pairwise comparisons (chronic drug versus control and chronic drug versus deprescribing) was used to determine reversibility. Dotted vertical and horizontal lines are log_2_(FC) threshold of ±0.58 (equivalent to 1.5‐ and 0.67‐FC). Additional hypothetical representative boxplots along with color‐coded regions are annotated in the scatterplot to depict the two different deprescribing outcomes: (i) reversible and (ii) irreversible. Note that, the boxplots only depict one directionality. Pearson’s correlation coefficient (*R*) and the corresponding *p*‐value were recorded. (b–e) Several representative boxplots (derived from polypharmacy) were presented to demonstrate the different deprescribing outcomes. (b) A reversible outcome occurs when the changes induced by chronic drug treatment are returned to control by deprescribing. (c) On the contrary, a “canonical” irreversible outcome is when the protein expression level of deprescribed group is similar to chronic drug treatment. (d) However, within this irreversible category, we also observed those deprescribing expression with high inter‐individual variability. (e) Additionally, deprescribing can also potentially produce novel effects that were not observed in the chronic drug treatment. Note that reversible and irreversible outcomes were determined by two pairwise comparisons (chronic drug versus control and chronic drug versus deprescribing) whereas novel deprescribing effects also considered deprescribing versus control comparison. * false discovery rate (FDR)‐adjusted *p* < 0.10, **p < 0.05, ***p < 0.01, ****p < 0.001. (f) Similar log_2_(FC) scatterplots were created for the five monotherapies, color‐coded accordingly.

**Figure S8.** Heatmap summary of deprescribing outcomes. Chronic drug effects (against control) were color‐coded based on log_2_(fold change [FC]) (red indicating upregulation and blue indicating downregulation). Only significant results were colored accordingly (*p* < 0.05 and >± 1.50 FC) and non‐significant comparisons were colored gray. Shapes indicate deprescribing outcomes: (i) reversible (circle), (ii) irreversible (cross), and novel deprescribing effect (triangle). Deprescribing outcomes were determined based on multiple pairwise comparisons (see methods and Figure S7). Proteins with no gene name were annotated with their UniProt ID.

**Figure S9.** Hepatic transcriptomics of deprescribing polypharmacy. (a) Volcano plot depicting polypharmacy versus polypharmacy deprescribed (left) and polypharmacy deprescribed versus control (right) in the transcriptome level. Vertical lines represent ±1.50 fold change (FC) and the horizontal lines depict raw *p* < 0.05 and false discovery rate (FDR)‐adjusted *p* < 0.05. (b) Scatterplot of RNA (*y*‐axis) and protein (*x*‐axis) log_2_(FC) derived from the corresponding pairwise comparisons with Pearson’s correlation analysis conducted on 5967 protein–transcript pairs. Correlation coefficients (*R*) and *p*‐values are shown. (c) Pathway‐level comparison as 2D annotation enrichment plot of log_2_(FC) enrichment score at the RNA (*y*‐axis) and protein (*x*‐axis). Gene ontology (GO) terms and Kyoto encyclopedia of genes and genomes (KEGG) pathways are color‐coded, and selected enrichment are highlighted. All enrichment data point depicted are significant enrichment (FDR <0.01) with dot sizes represents *p*‐value.

**Figure S10.** Weighted gene co‐expression network analysis (WGCNA) network construction. (a) Scale‐free fit index (left) and mean connectivity (right) of various soft‐threshold powers. Horizontal dotted line depicts scale‐free fit index of 0.85. Selected soft‐threshold power of 5 is indicated by red. (b) Cluster dendrogram of the proteome using unsupervised hierarchical clustering followed by dynamic tree cutting, which resulted in 22 co‐expressed modules. (c) Number of proteins assigned in each module. Modules were color‐coded and ordered in a descending manner. Module M0 (gray) is for unassigned proteins.

**Figure S11.** Module–trait relationship analysis of weight, serum biochemistry, and serum cytokine levels. Heatmap summarizing module–trait relationship analysis between the 23 co‐expressed protein modules (columns) and different clinically relevant phenotypes (rows). Degree of positive (red) and negative (blue) correlation are shown, with asterisks representing significance (*p < 0.05, **p < 0.01, ***p < 0.001) based on biweight midcorrelation analysis for weights, serum biochemistry, and serum cytokine levels. Based on GO and KEGG enrichment analysis, selected functional categories of each module were noted at the bottom of the heatmap with their corresponding color codes displayed at the top of the heatmap.

**Figure S12.** Comparing module eigenprotein across treatment groups and expression of hub proteins. Modules of interest were modules that displayed association(s) with any of the geriatric behavioral outcomes, which include (a) M9, (b) M16, and (c) M22. One‐way ANOVA statistics, including degrees of freedom, *F* statistics, *p*‐value, and effect size (*η* ^2^) were recorded. No statistical significance was detected. (d) Heatmap after hierarchical clustering of the hub proteins across all. Unsupervised hierarchical clustering was conducted with Euclidean distance and complete linkage in which six row and two column clusters were marked. Column is annotated with drug treatment and deprescribing grouping, while row annotation is based on the six co‐expressed modules. Heatmap color code is based on row *z*‐score scaled with red indicating higher abundance and blue indicating lower.

## Data Availability

Proteomics raw files can be accessed through the ProteomeXchange/PRIDE (PXD049469) (https://www.ebi.ac.uk/pride/archive/projects/PXD049469). The transcriptomics data are deposited at the NIH SRA (PRJNA1165228) (http://www.ncbi.nlm.nih.gov/bioproject/1165228). [Correction added on 22 November 2024, after first online publication: The Open Research/Data Availability Statement have been updated in this version.]
