# Supplementary figures and images for: Chronic polypharmacy, monotherapy, and deprescribing: Understanding complex effects on the hepatic proteome of aging mice
Source: Aging Cell. 2024 Oct 27;24(1):e14357. doi: 10.1111/acel.14357 (PMC11709111; doi:10.1111/acel.14357)

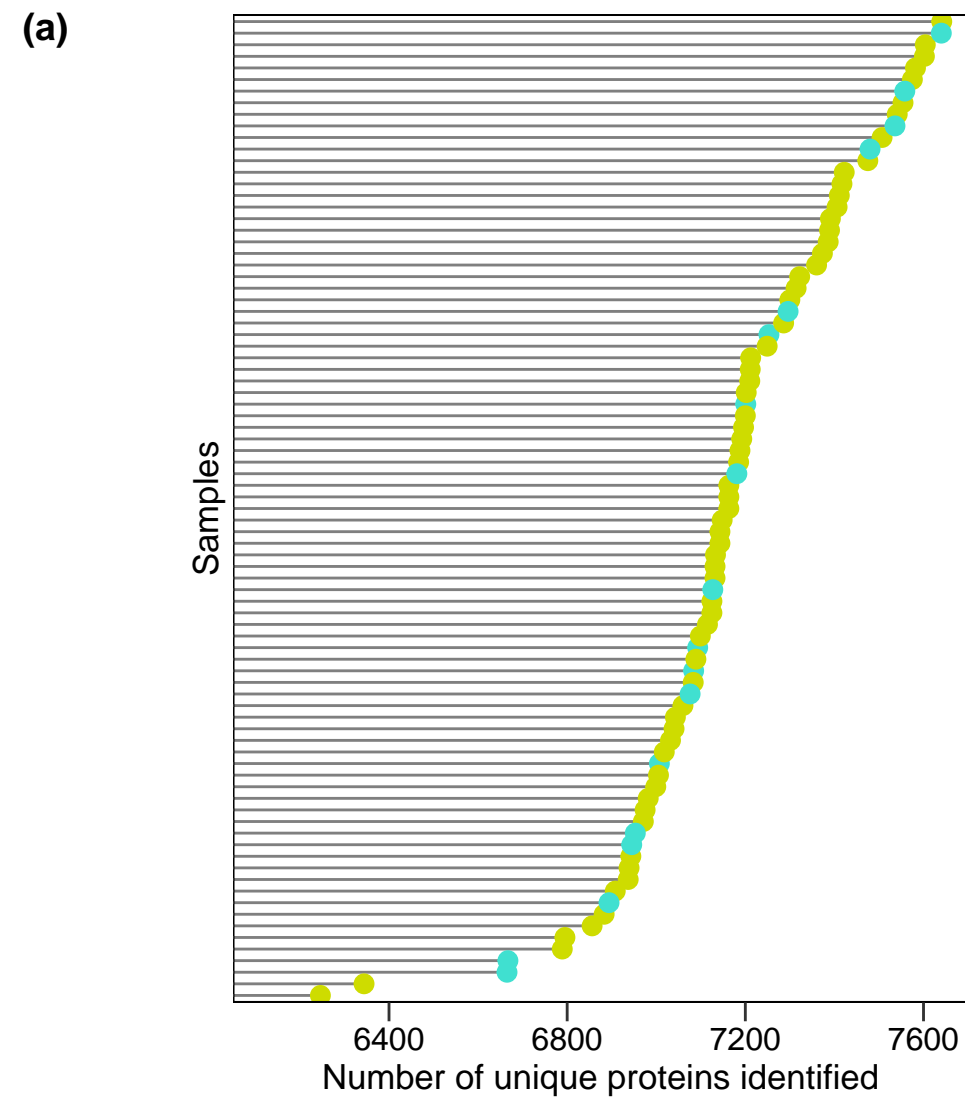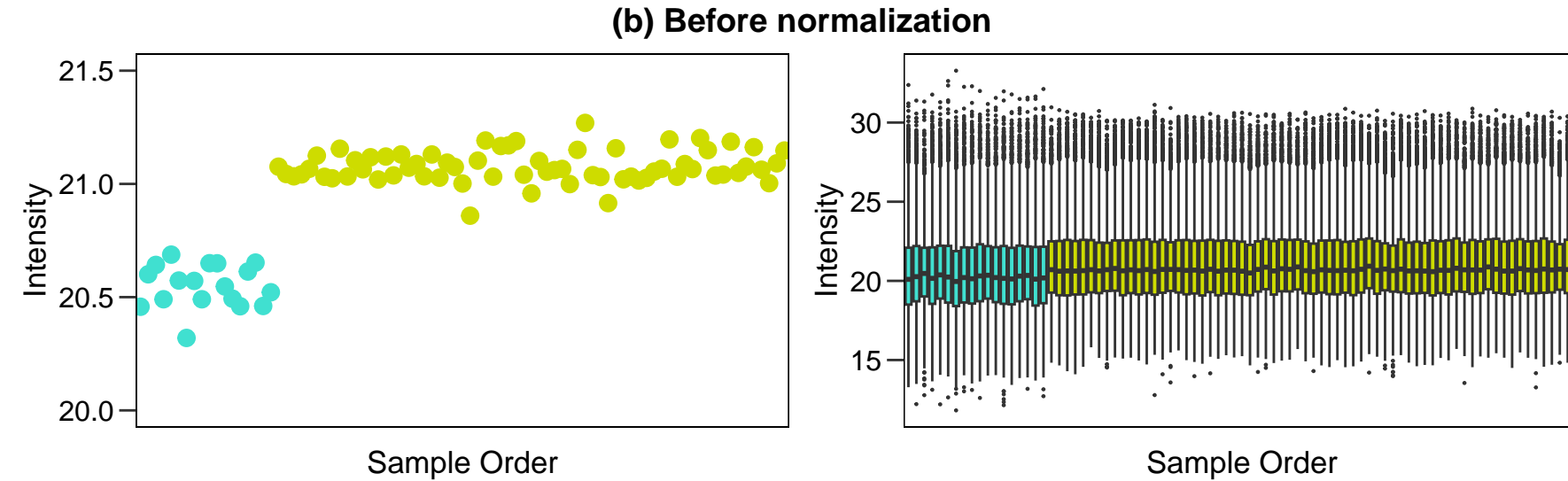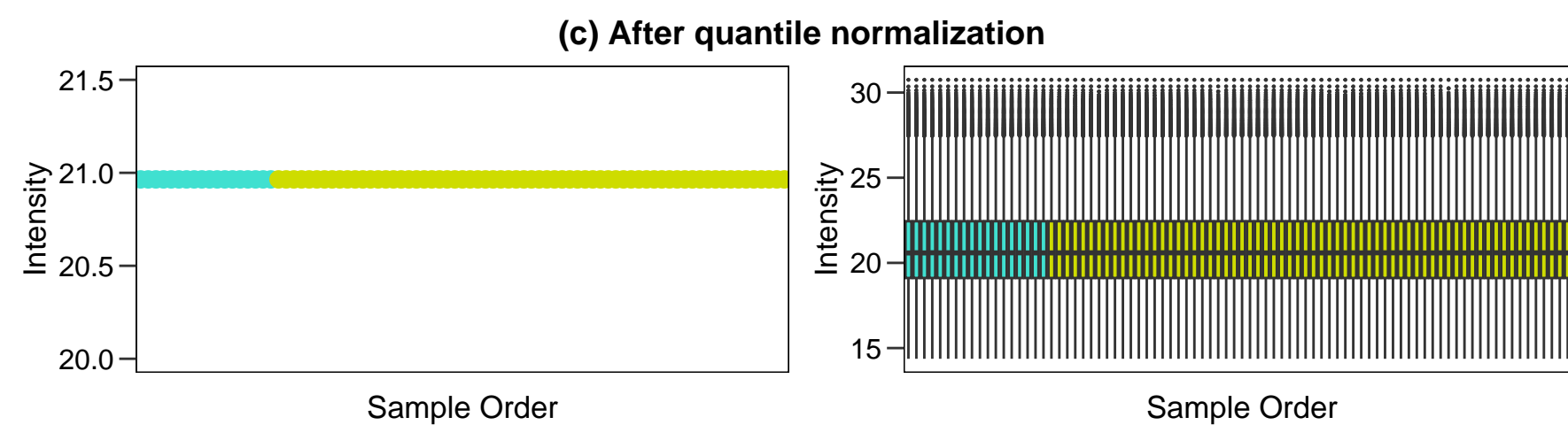

● MS batch 1 ● MS batch 2

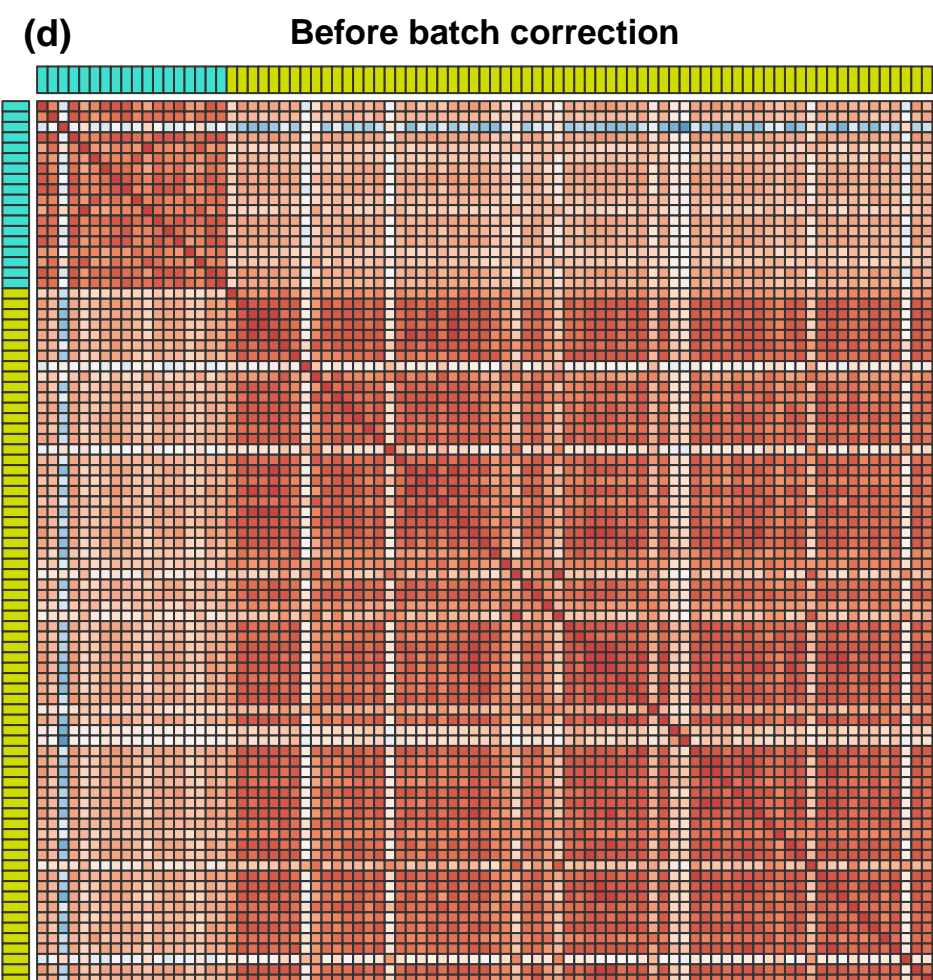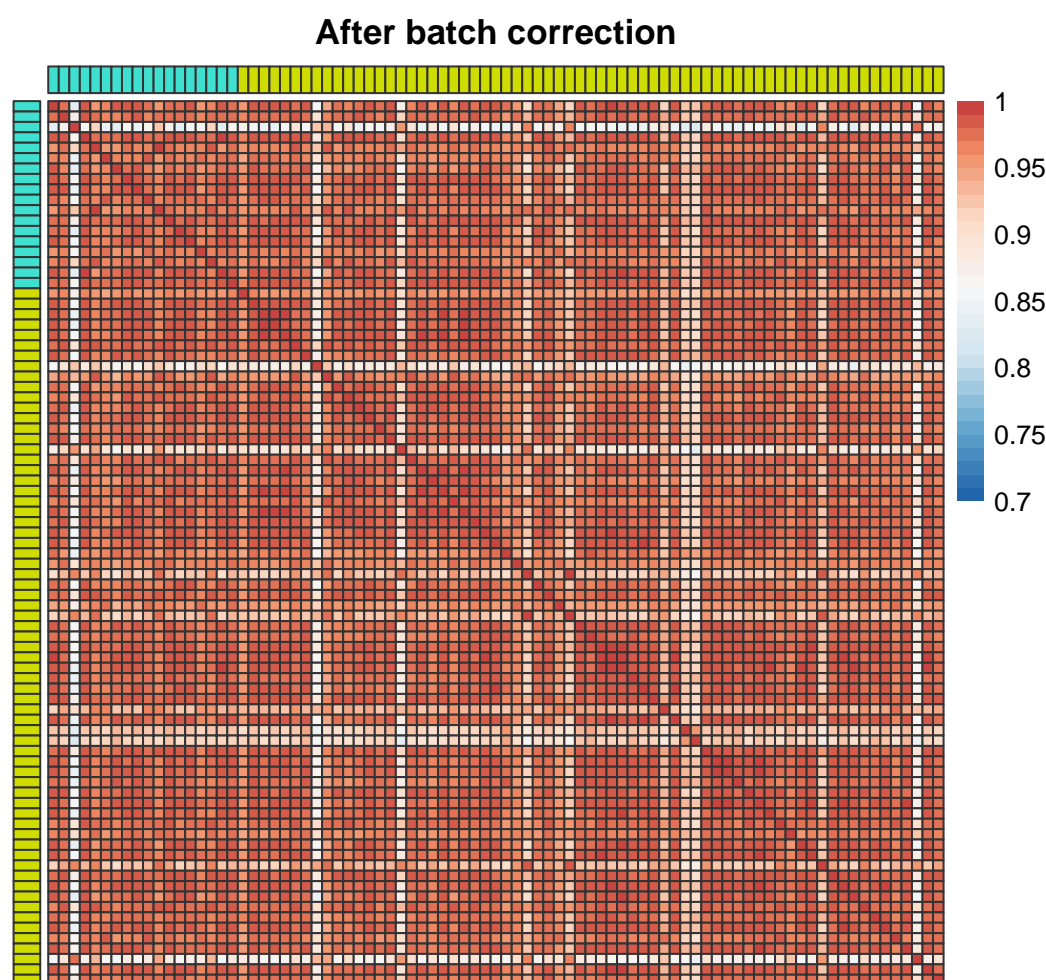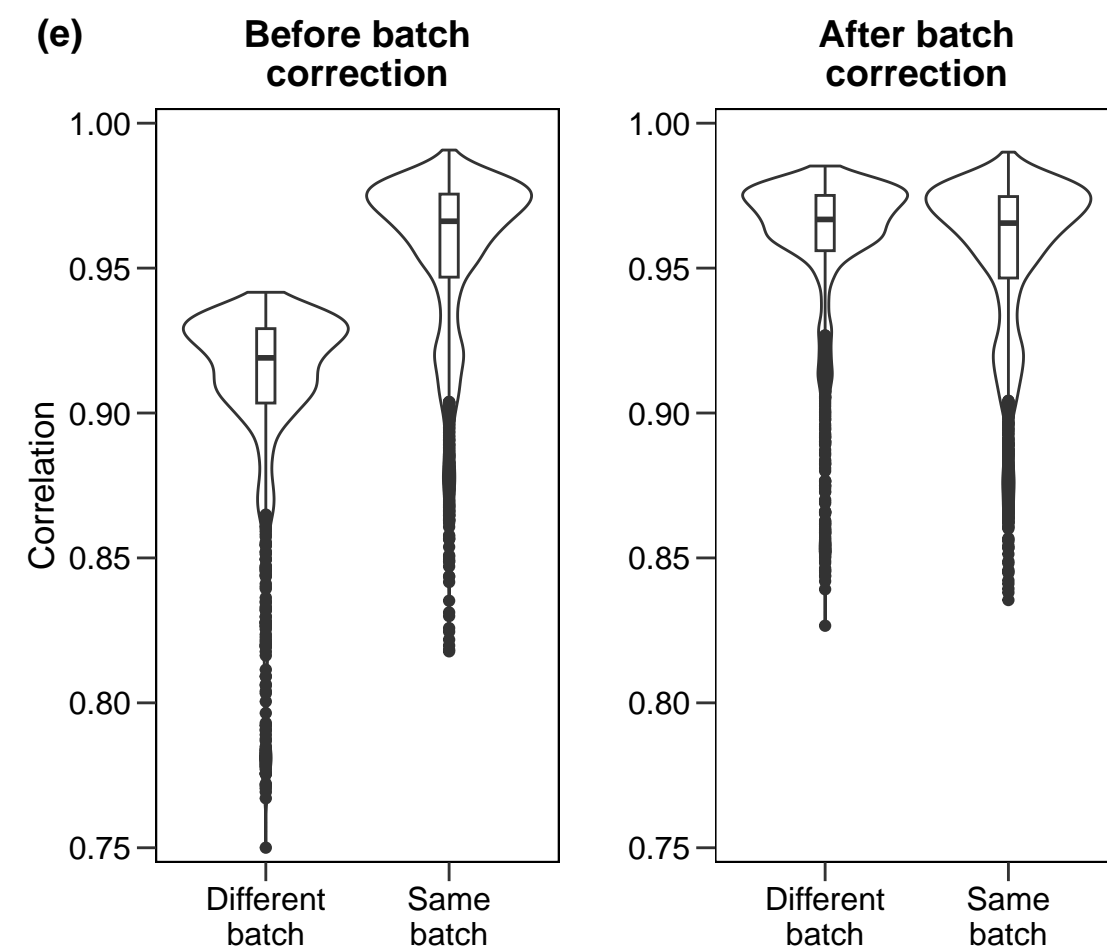

Supplement: Supplementary file 2 — Figure S1. Proteomics preprocessing strategies. (a) Number of proteins quantified in each sample (peptide and protein false discovery rate [FDR] <1%). Mean intensity and boxplot intensity of each sample (b) before and (c) after quantile normalization, color‐coded based on batch. (d) Correlation matrix between samples before and after batch effect correction using ComBat algorithm. (e) Violin plot depicting Pearson’s correlation coefficient between‐batch and within‐batch. [file ACEL-24-e14357-s010.pdf]

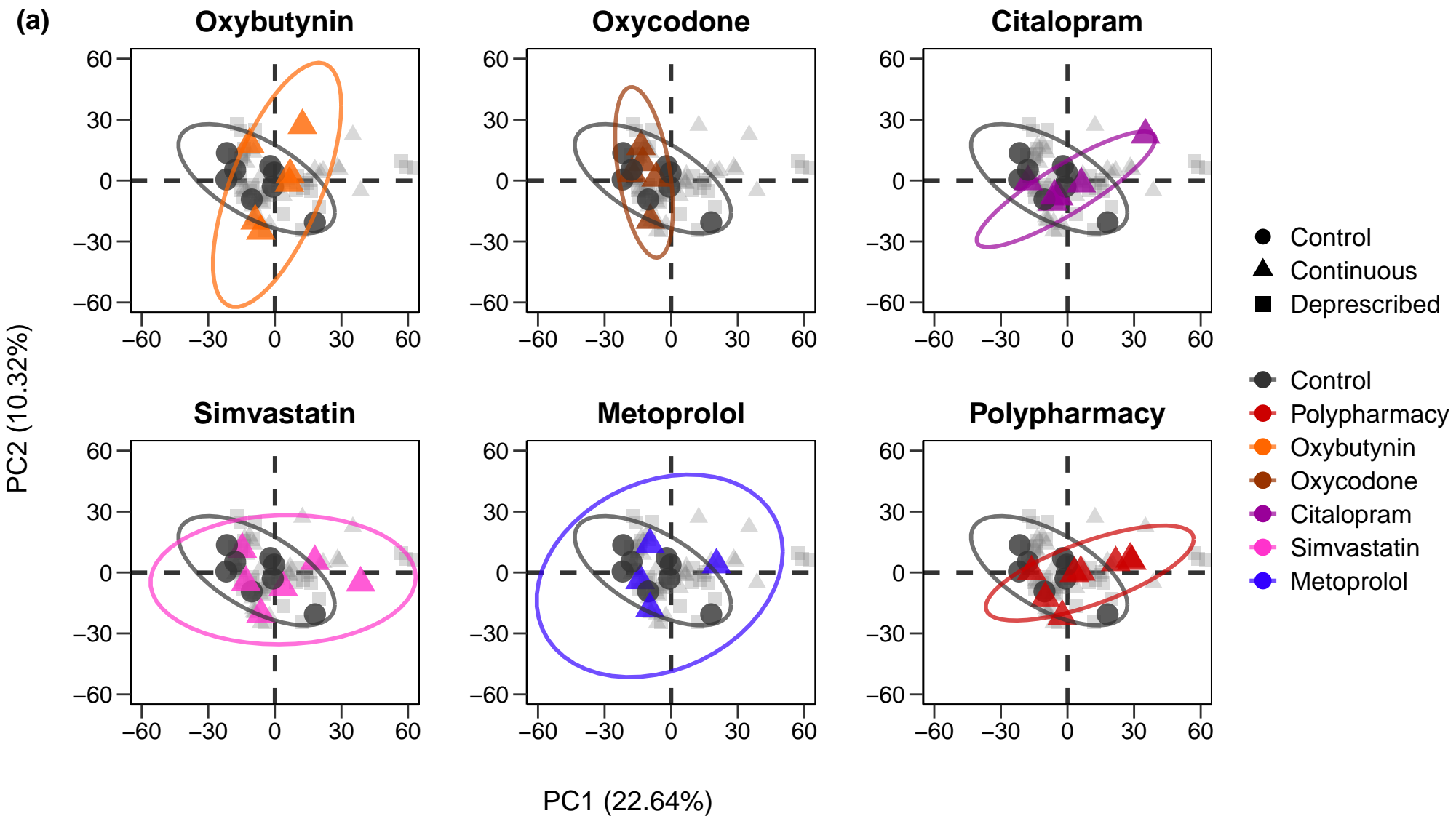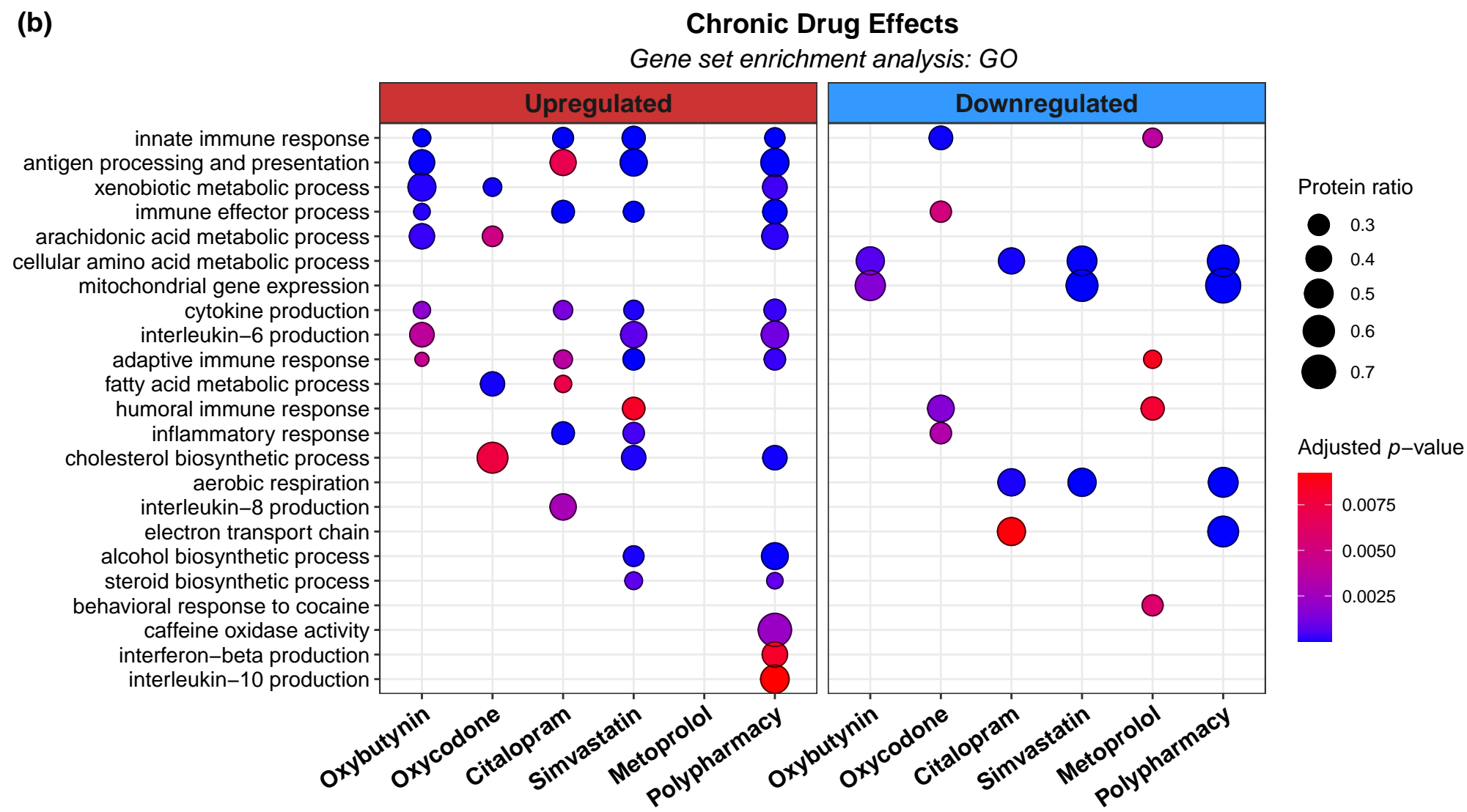

Supplement: Supplementary file 3 — Figure S2. Principal component analysis (PCA) and enrichment analysis of chronic monotherapy and polypharmacy. (a) PCA comparing chronic (continuous) drug effects. Samples are color‐coded by drug treatments and shape indicates chronic (triangle) or deprescribed (square) drug treatment. Here, only chronic drug treatment groups and control cluster ellipses (95% confidence) are depicted, with deprescribed samples uncolored. The explained variance (%) is denoted in brackets in the axis titles. (b) Dot plot showing gene set enrichment analysis (GSEA) of selected gene ontology (GO) pathways enriched in chronic drug treatment compared with control, with size of dots proportional to protein ratio and colors representing false discovery rate (FDR)‐adjusted p‐value. [file ACEL-24-e14357-s009.pdf]

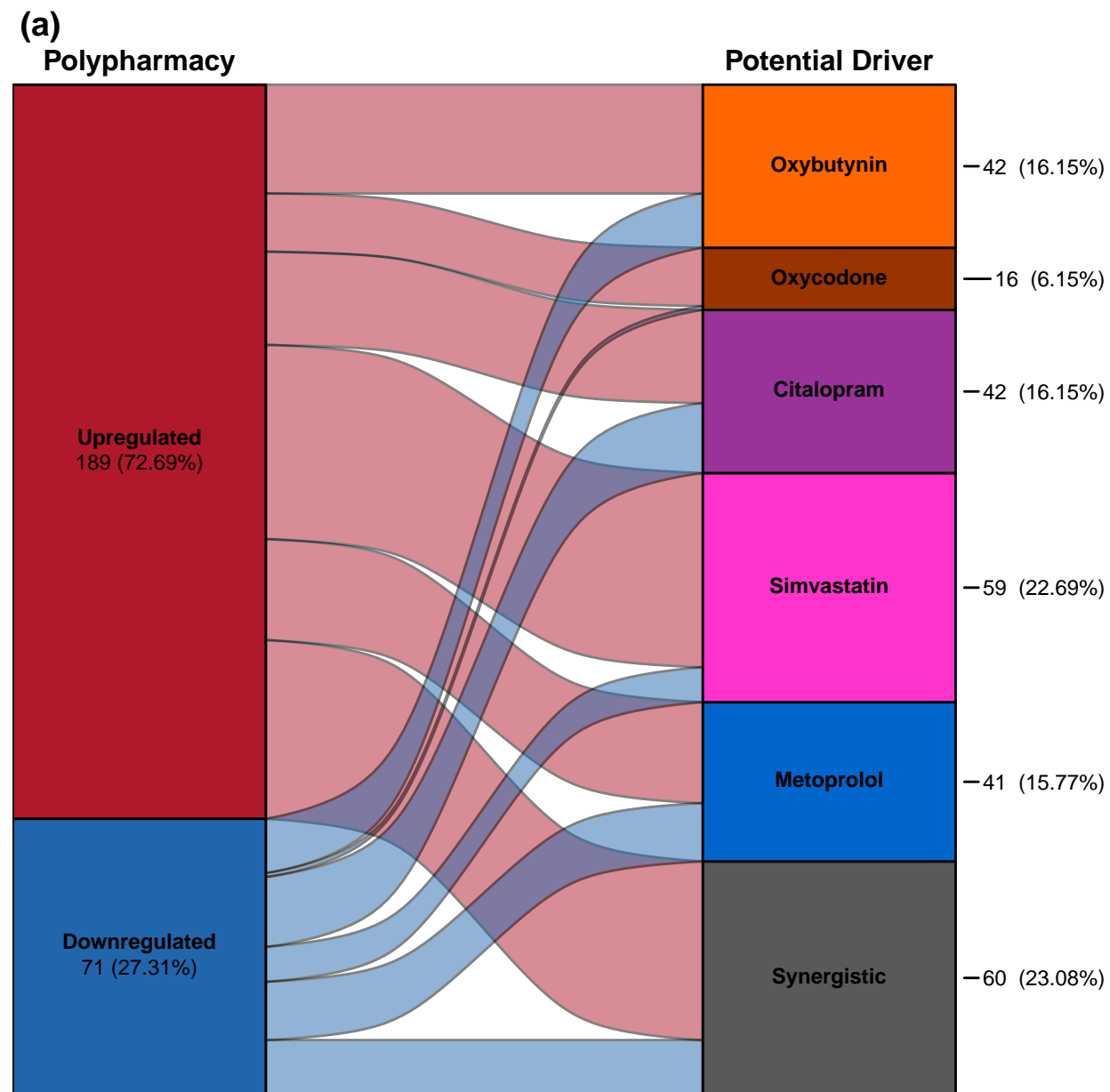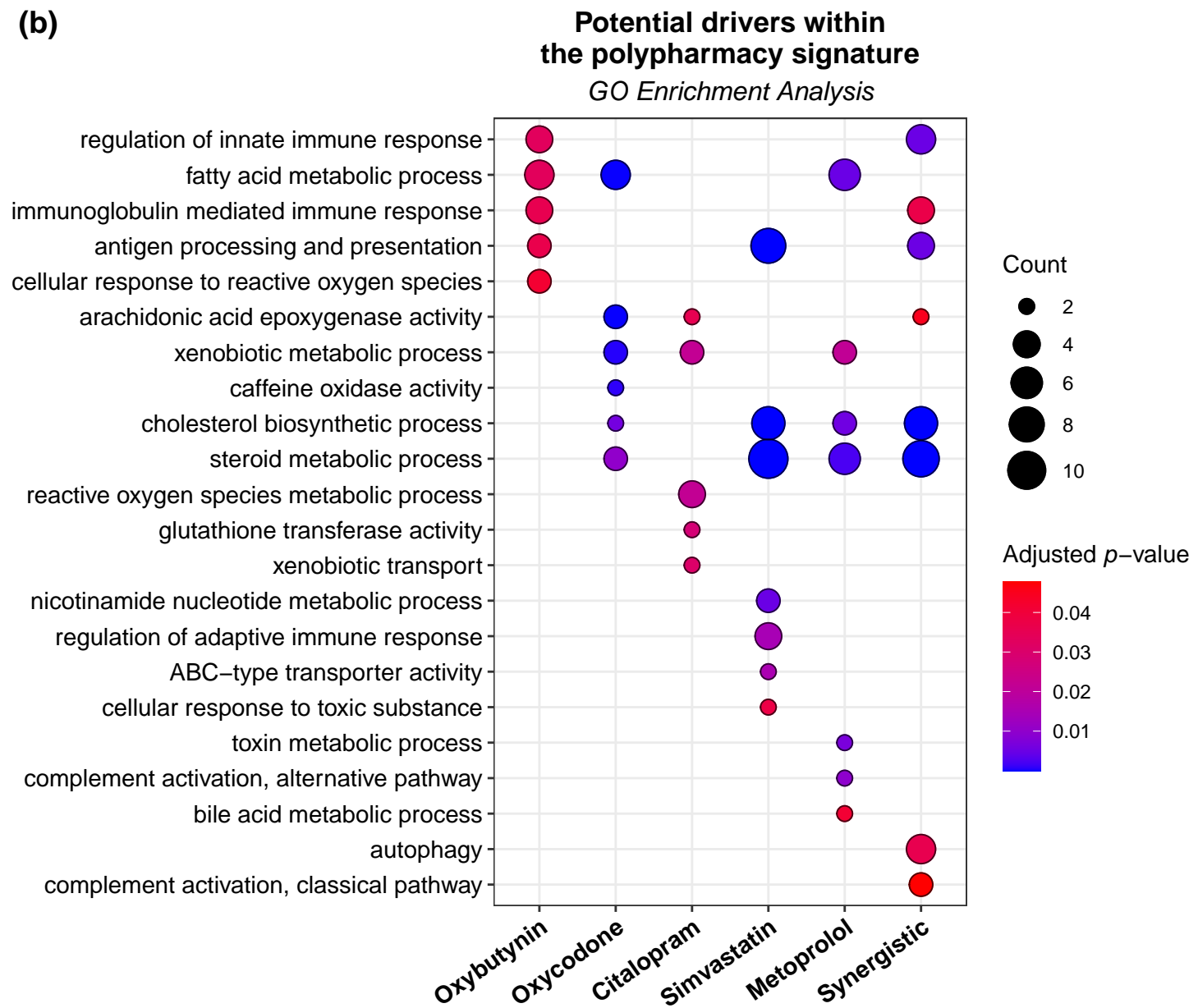

Supplement: Supplementary file 4 — Figure S3. Identifying dominant drug driver in the polypharmacy regimen with synergism restriction. (a) Similar to Figure 2c, the alluvial plot depicts the potential drug driver of the polypharmacy regimen with the additional filter in which only sub‐additive effects were considered for single drug driver analysis, and the rest are attributed to unique synergistic polypharmacy effect. Protein counts and proportion (%) were shown. (b) Dot plot showing selected gene ontology (GO) pathways enriched in the five “dominant drug” clusters along with the synergistic polypharmacy cluster within the polypharmacy signature, with size of dots proportional to protein counts and colors representing false discovery rate (FDR)‐adjusted p‐value. [file ACEL-24-e14357-s007.pdf]

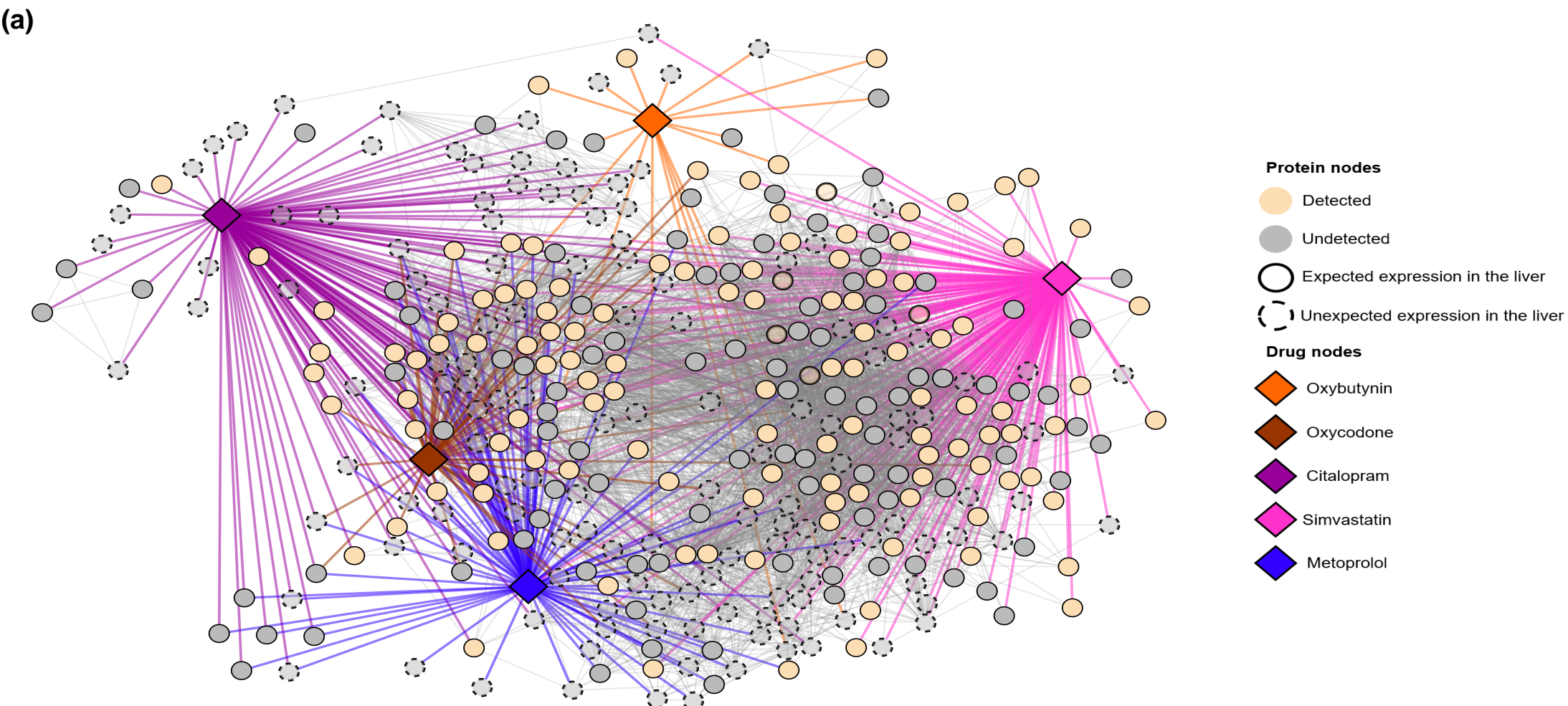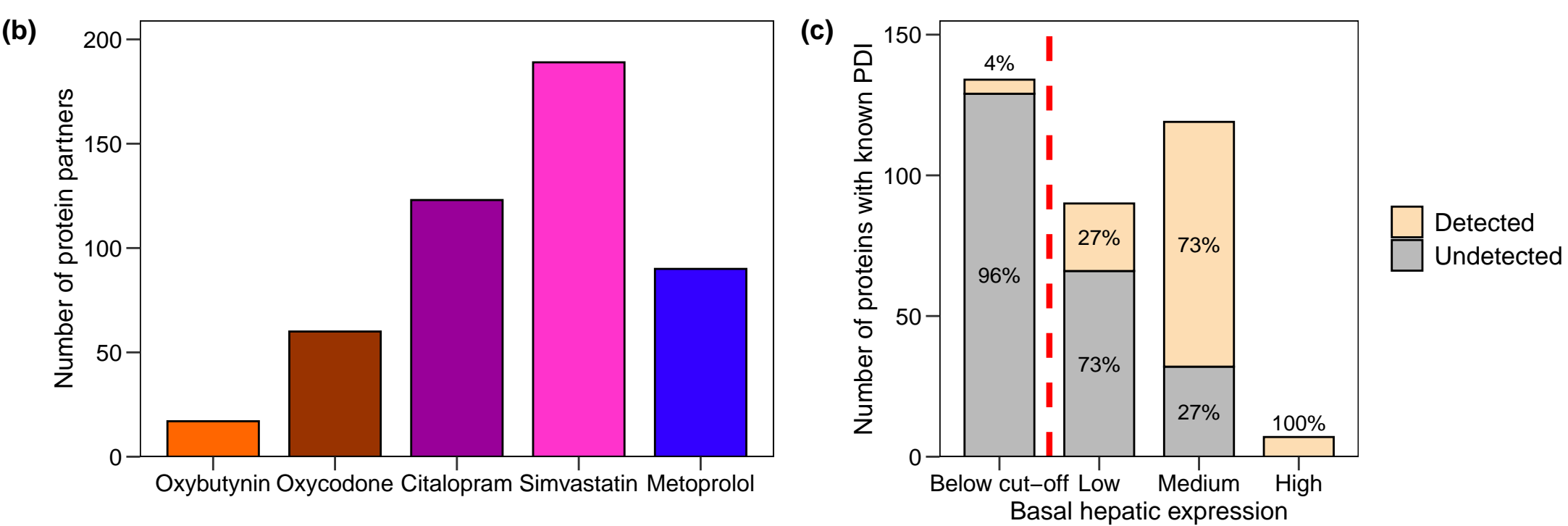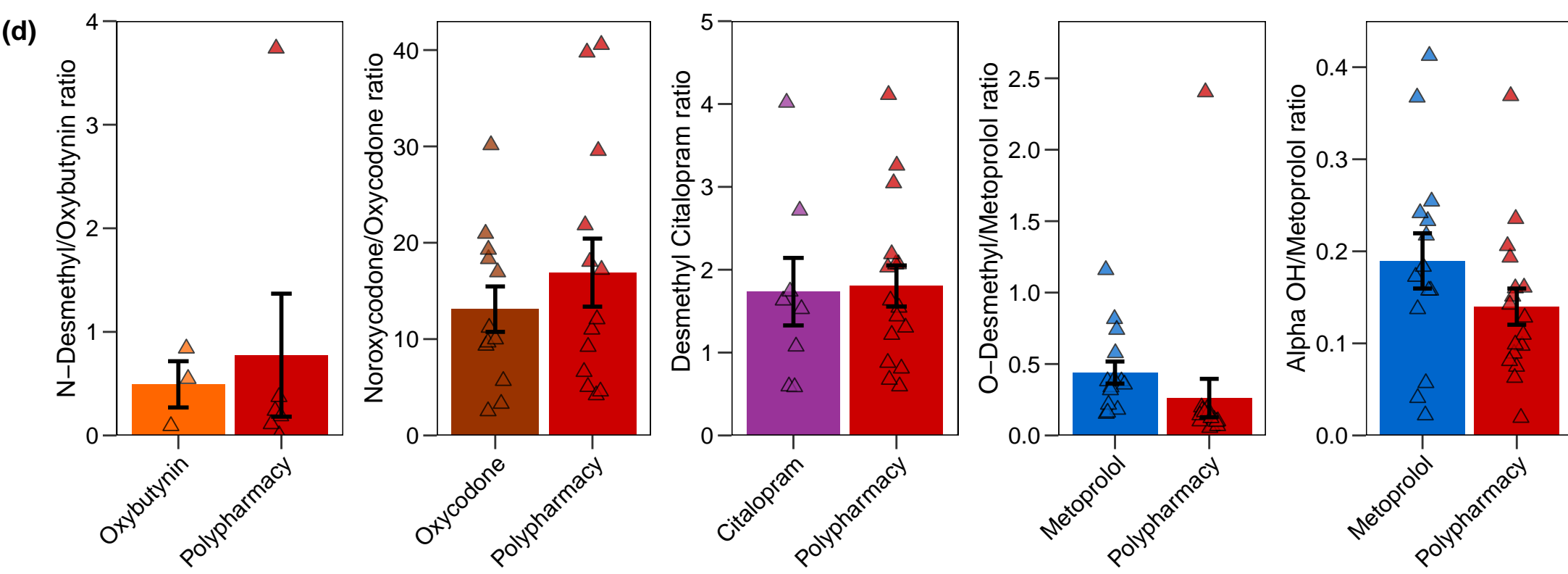

Supplement: Supplementary file 5 — Figure S4. Properties of protein–drug interaction (PDI) network in a murine hepatic system and serum metabolite‐to‐parent ratio. (a) Complete Search tool for Interacting Chemicals (STITCH)‐derived PDI network consisting of 361 protein nodes (circle) and five drug nodes (rhombus) with 3941 edges. Drug nodes and its respective interactions with proteins were color‐coded accordingly. Protein–protein interaction edges were colored as gray. Basal hepatic expression, based on Mouse Genome Informatics Gene eXpression Database (MGI GXD) RNA‐seq data, was annotated with node borders wherein solid line indicates expected expression while dotted line indicates not. Proteins identified in the hepatic proteome dataset were color‐coded as yellow. (b) Barplot of number of direct interactions each drug is known to be involved with. (c) Basal expression of the PDI proteins in C57BL/6J male murine liver. Categories were determined from RNA‐seq data (normalized as transcript per million [TPM]) with default setting: (i) below cutoff (0.00–0.49 TPM), (ii) low (0.50–10.00 TPM), (iii) medium (10.01–1000.00 TPM), and (iv) high (>1000.00 TPM). Proteins that classified as low, medium, or high expression were deemed basally expressed in the liver (bars on the right of the red dotted vertical line). Bars were stacked based on whether the proteins were detected in the proteomics dataset annotated as proportion (%) of total basal hepatic expression per expression categories. (d) Serum levels of drug metabolite‐to‐parent ratios from 24‐month‐old mice comparing polypharmacy against the corresponding monotherapies. Data is represented as mean ± SEM. Two‐tailed Student’s t test was conducted and no significance was identified. [file ACEL-24-e14357-s008.pdf]

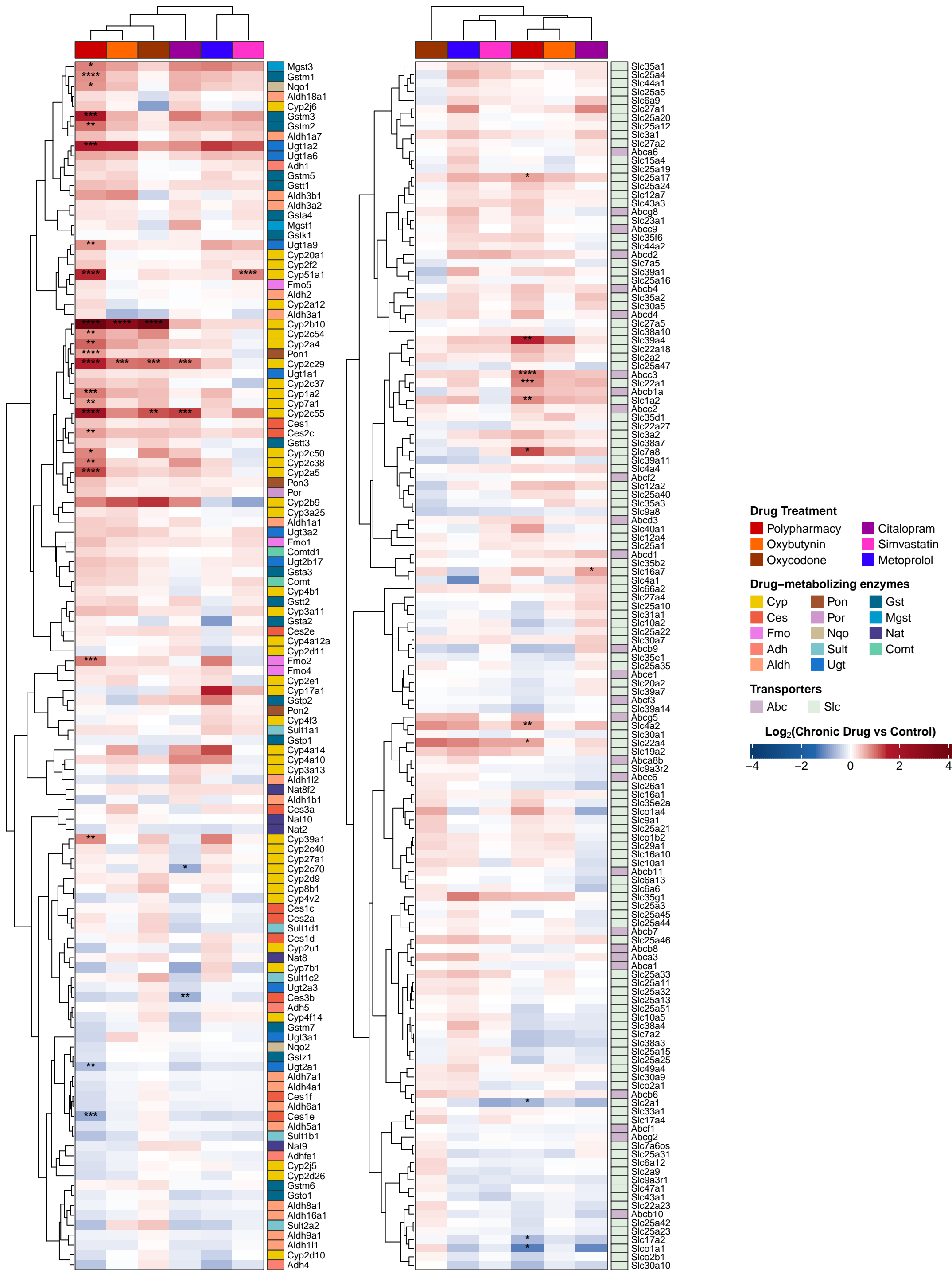

Supplement: Supplementary file 6 — Figure S5. Probing for drug‐metabolizing enzymes and transporters. Log2(fold change [FC]) heatmap of drug‐metabolizing enzymes (left) and transporters (right) between chronic drug treatment versus control. Heatmap was color‐coded (red indicating upregulation and blue indicating downregulation) and annotated with statistical significance: * false discovery rate (FDR)‐adjusted p < 0.10, **p < 0.05, ***p < 0.01, ****p < 0.001. Unsupervised hierarchical clustering was performed with Euclidean distance and complete linkage. Drug‐metabolizing and transporter classes were also annotated. Cyp: cytochrome P450; Ces: carboxylesterase; Fmo: flavin‐containing monooxygenase; Adh: alcohol dehydrogenase; Aldh: aldehyde dehydrogenase; Pon: paraoxonase; Por: P450 oxidoreductase; Nqo: NADPH‐quinone oxidoreductase; Sult: sulfotransferase; Ugt: UDP‐glucuronosyltransferase; Gst: glutathione S‐transferase; Mgst: microsomal glutathione S‐transferases; Nat: N‐acetyltransferase; Comt: catechol‐O‐methyltransferase; Abc: ATP‐binding cassette transporter; Slc: solute carrier transporter. [file ACEL-24-e14357-s005.pdf]

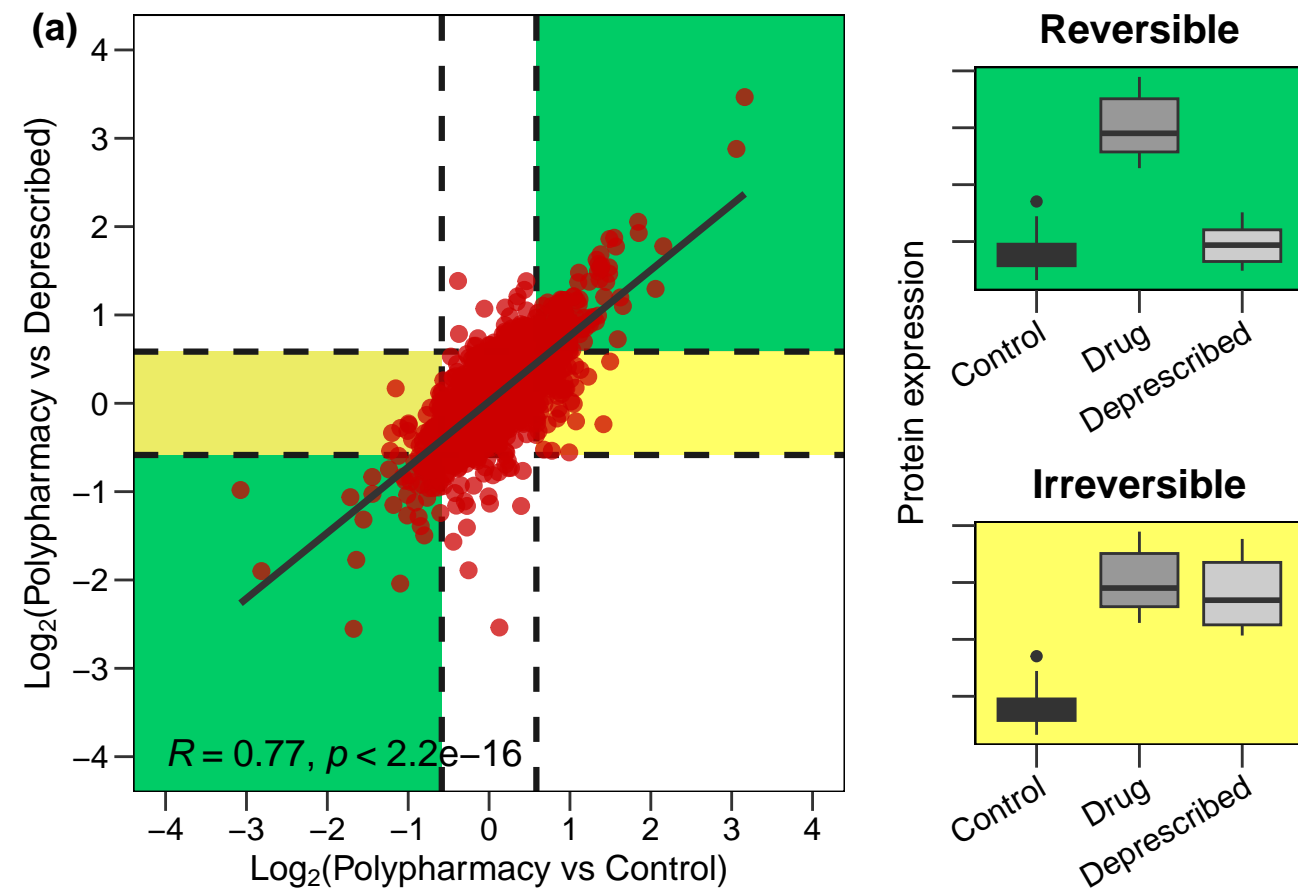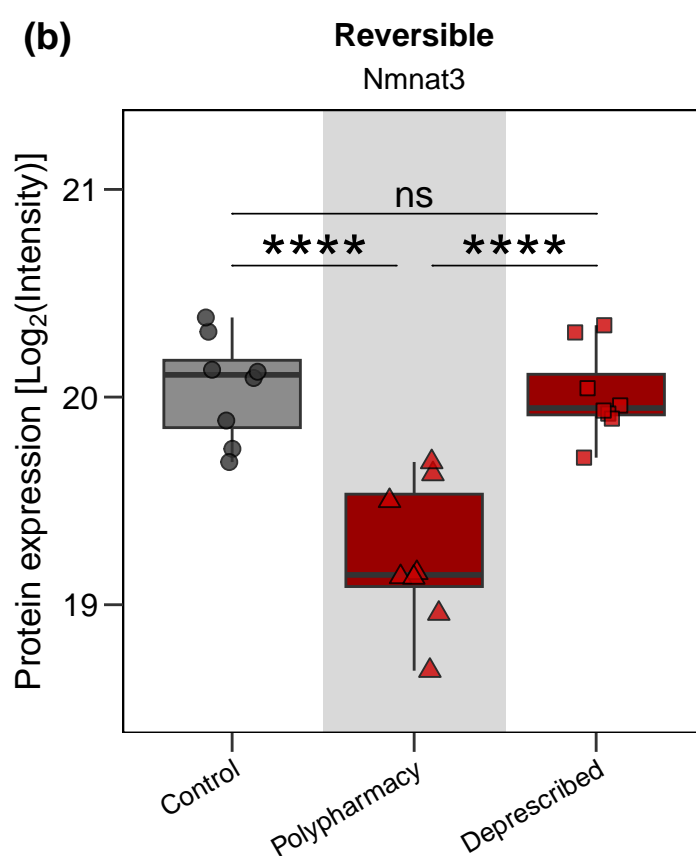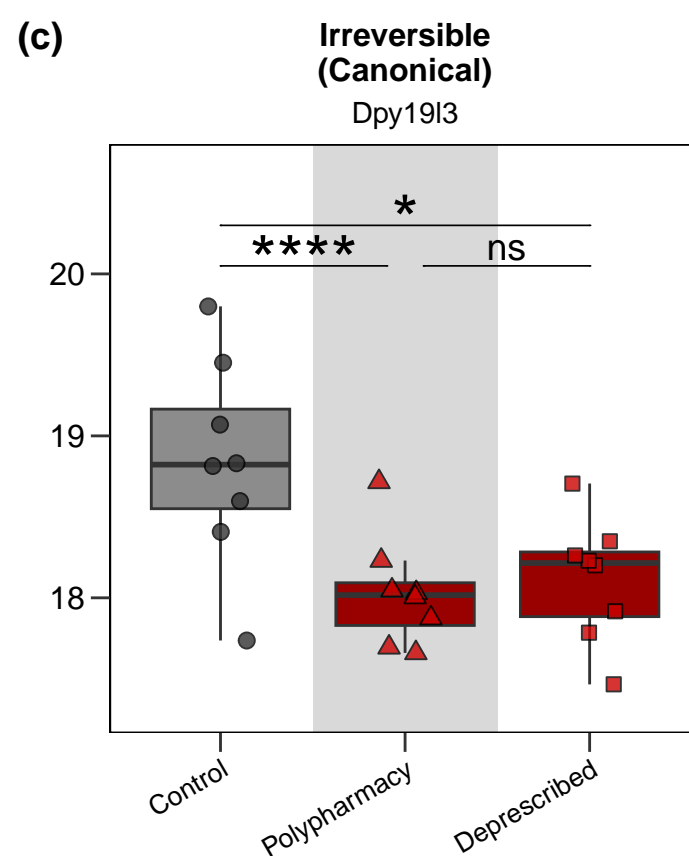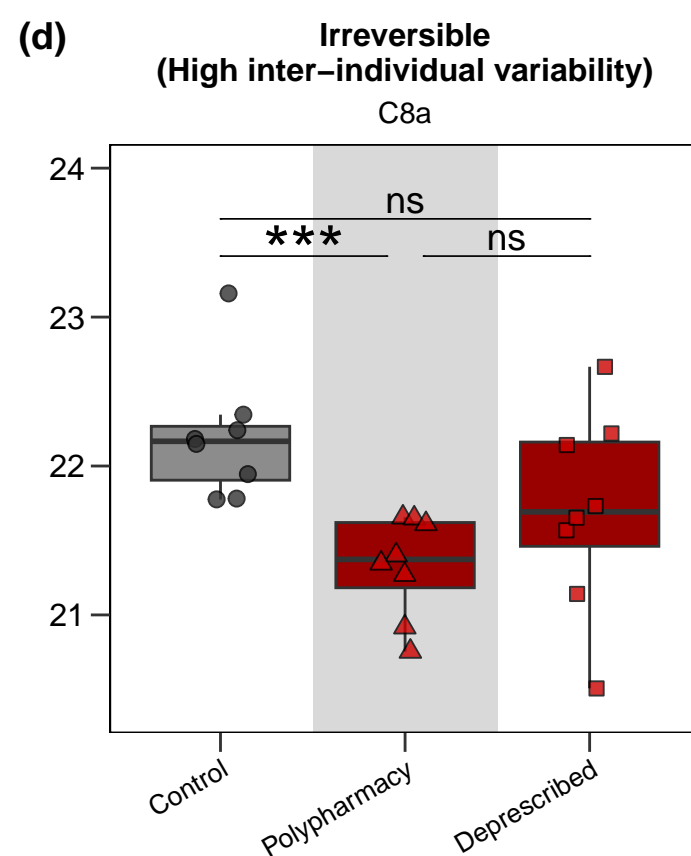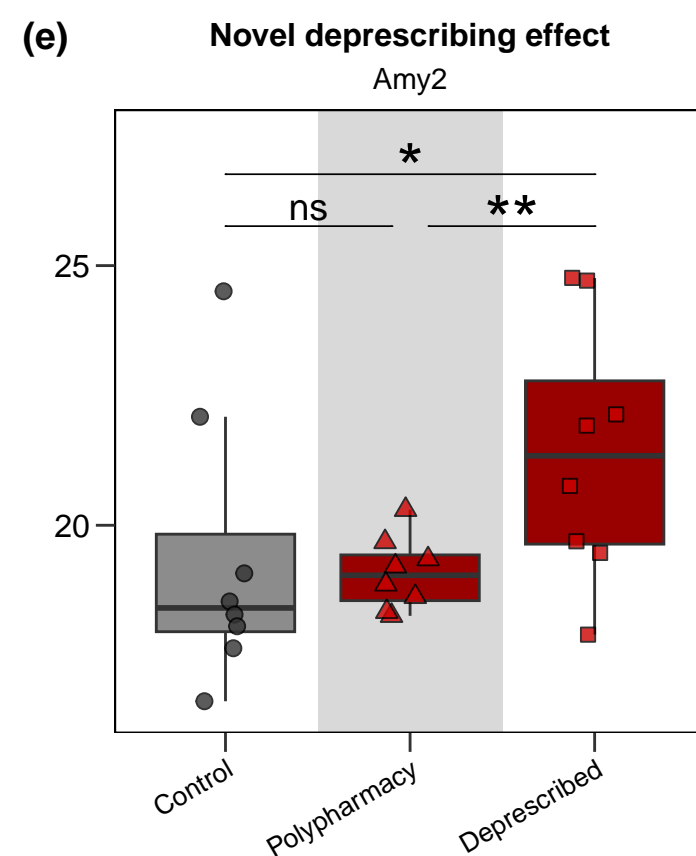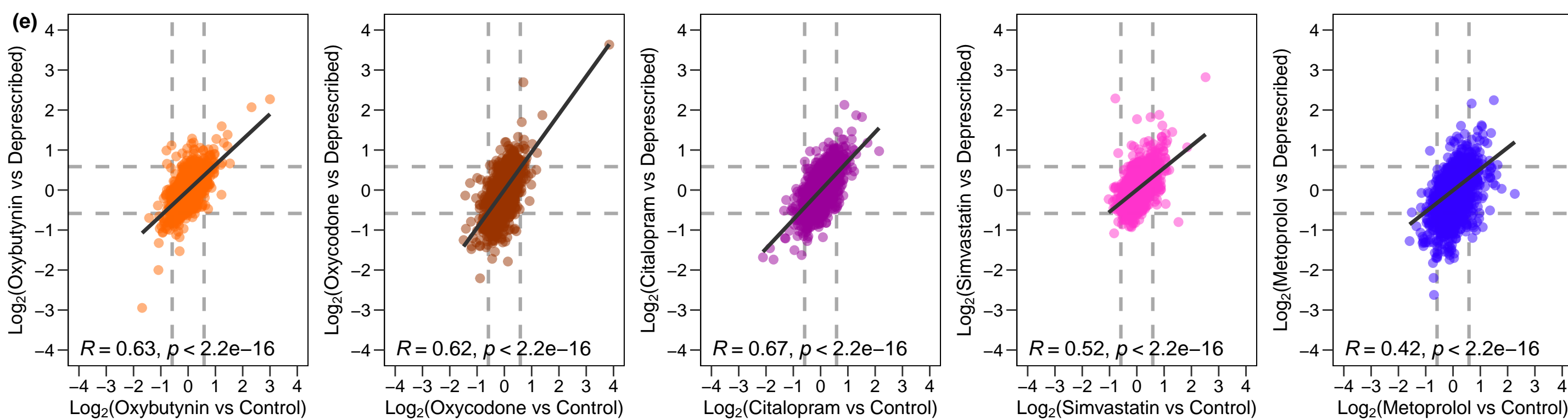

Supplement: Supplementary file 8 — Figure S7. Log2(fold change [FC]) scatterplots, representative boxplots, and statistical requirements for deprescribing outcomes. (a) Considering chronic drug treatment, its respective deprescribing group, and control, a log2(FC) scatterplot of two pairwise comparisons (chronic drug versus control and chronic drug versus deprescribing) was used to determine reversibility. Dotted vertical and horizontal lines are log2(FC) threshold of ±0.58 (equivalent to 1.5‐ and 0.67‐FC). Additional hypothetical representative boxplots along with color‐coded regions are annotated in the scatterplot to depict the two different deprescribing outcomes: (i) reversible and (ii) irreversible. Note that, the boxplots only depict one directionality. Pearson’s correlation coefficient (R) and the corresponding p‐value were recorded. (b–e) Several representative boxplots (derived from polypharmacy) were presented to demonstrate the different deprescribing outcomes. (b) A reversible outcome occurs when the changes induced by chronic drug treatment are returned to control by deprescribing. (c) On the contrary, a “canonical” irreversible outcome is when the protein expression level of deprescribed group is similar to chronic drug treatment. (d) However, within this irreversible category, we also observed those deprescribing expression with high inter‐individual variability. (e) Additionally, deprescribing can also potentially produce novel effects that were not observed in the chronic drug treatment. Note that reversible and irreversible outcomes were determined by two pairwise comparisons (chronic drug versus control and chronic drug versus deprescribing) whereas novel deprescribing effects also considered deprescribing versus control comparison. * false discovery rate (FDR)‐adjusted p < 0.10, **p < 0.05, ***p < 0.01, ****p < 0.001. (f) Similar log2(FC) scatterplots were created for the five monotherapies, color‐coded accordingly. [file ACEL-24-e14357-s004.pdf]

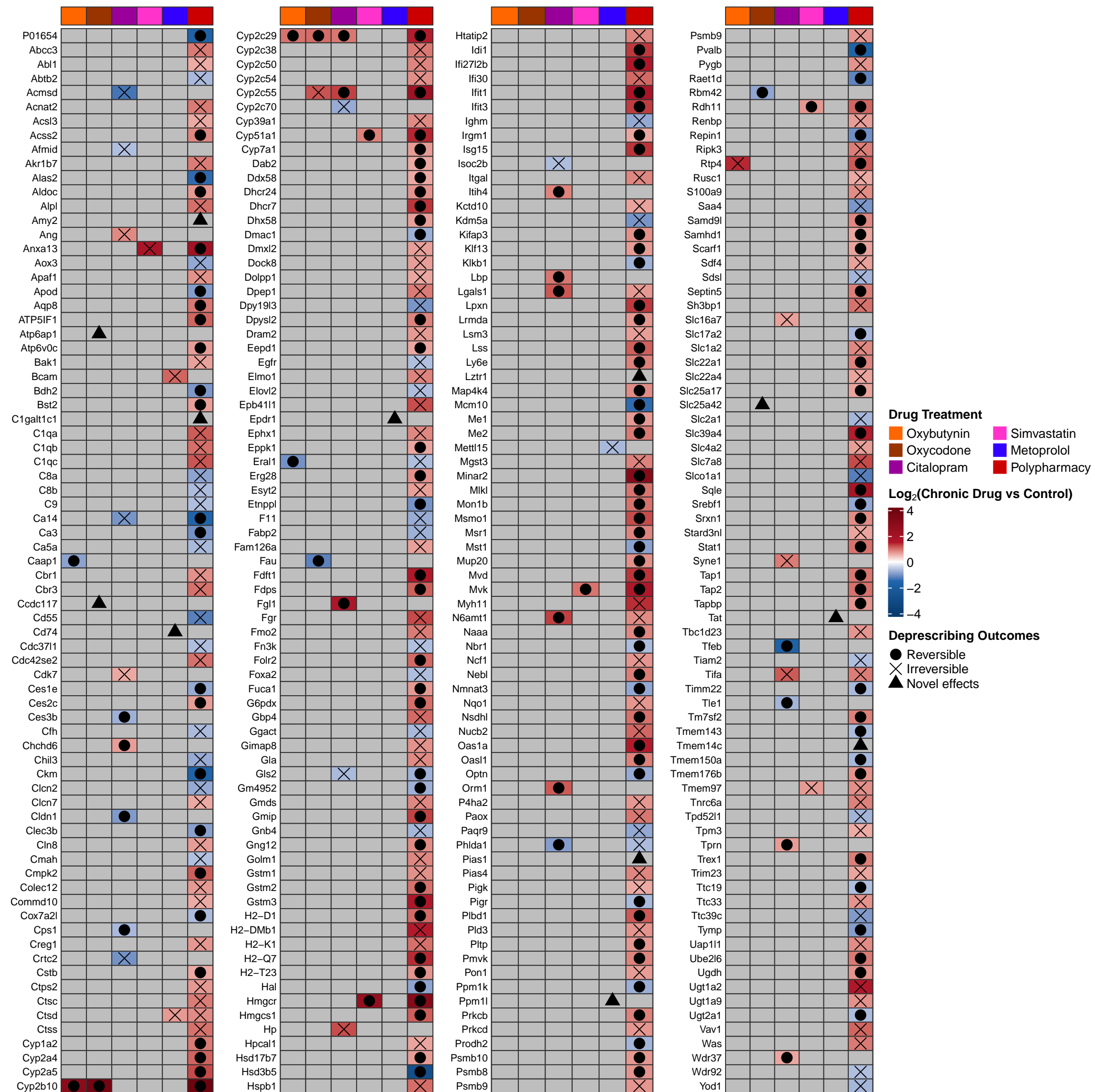

Supplement: Supplementary file 9 — Figure S8. Heatmap summary of deprescribing outcomes. Chronic drug effects (against control) were color‐coded based on log2(fold change [FC]) (red indicating upregulation and blue indicating downregulation). Only significant results were colored accordingly (p < 0.05 and >± 1.50 FC) and non‐significant comparisons were colored gray. Shapes indicate deprescribing outcomes: (i) reversible (circle), (ii) irreversible (cross), and novel deprescribing effect (triangle). Deprescribing outcomes were determined based on multiple pairwise comparisons (see methods and Figure S7). Proteins with no gene name were annotated with their UniProt ID. [file ACEL-24-e14357-s013.pdf]

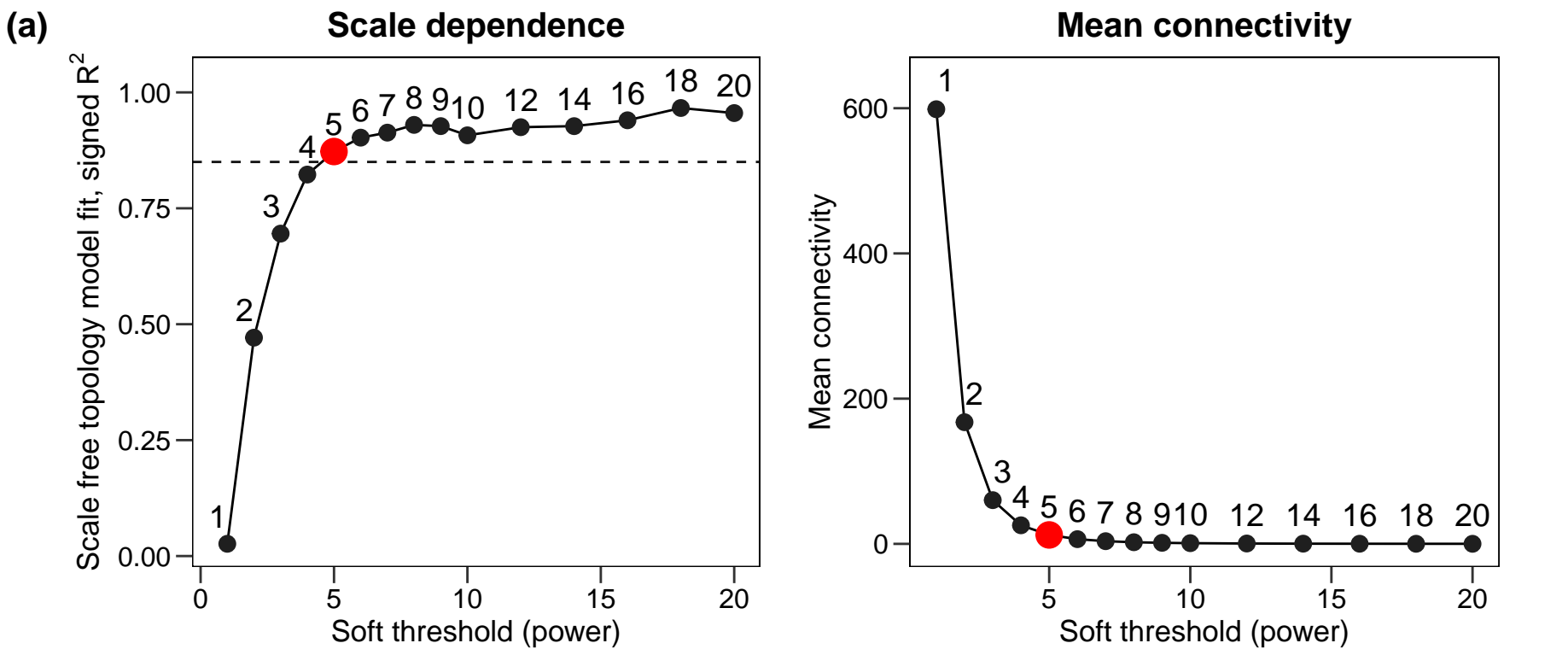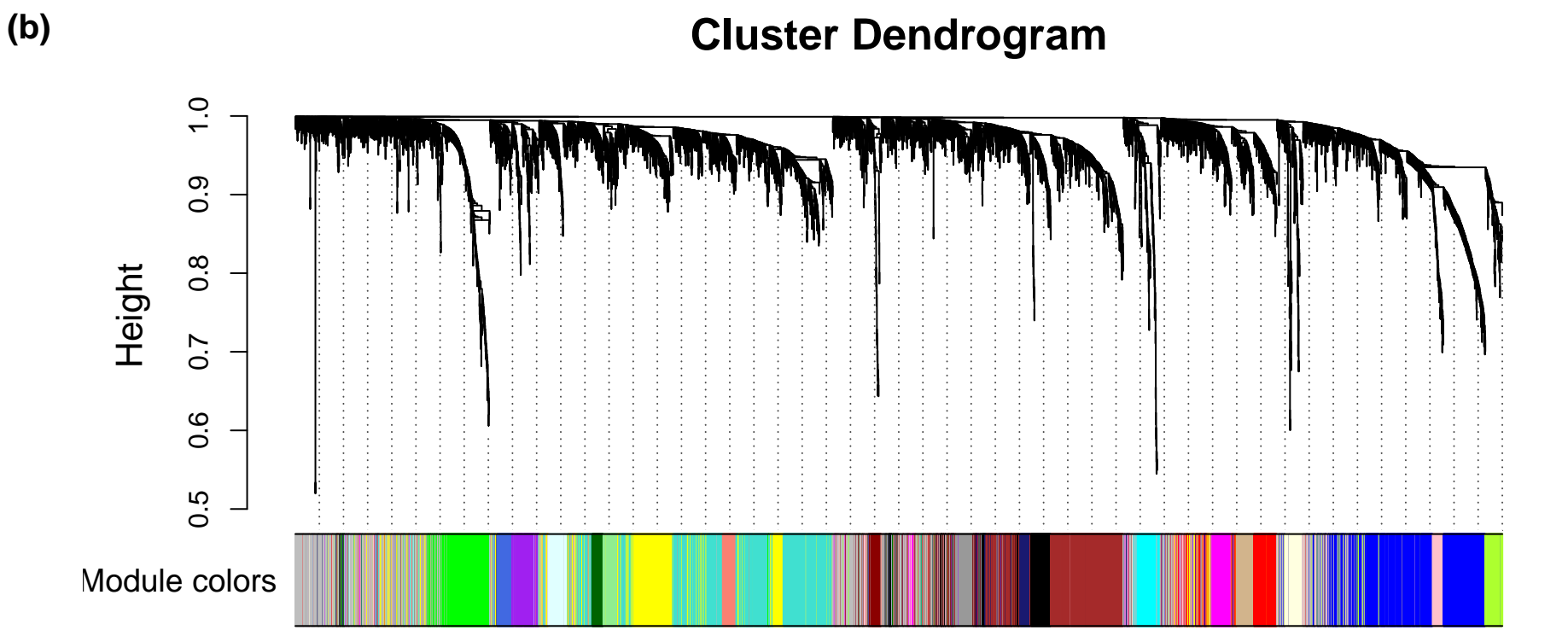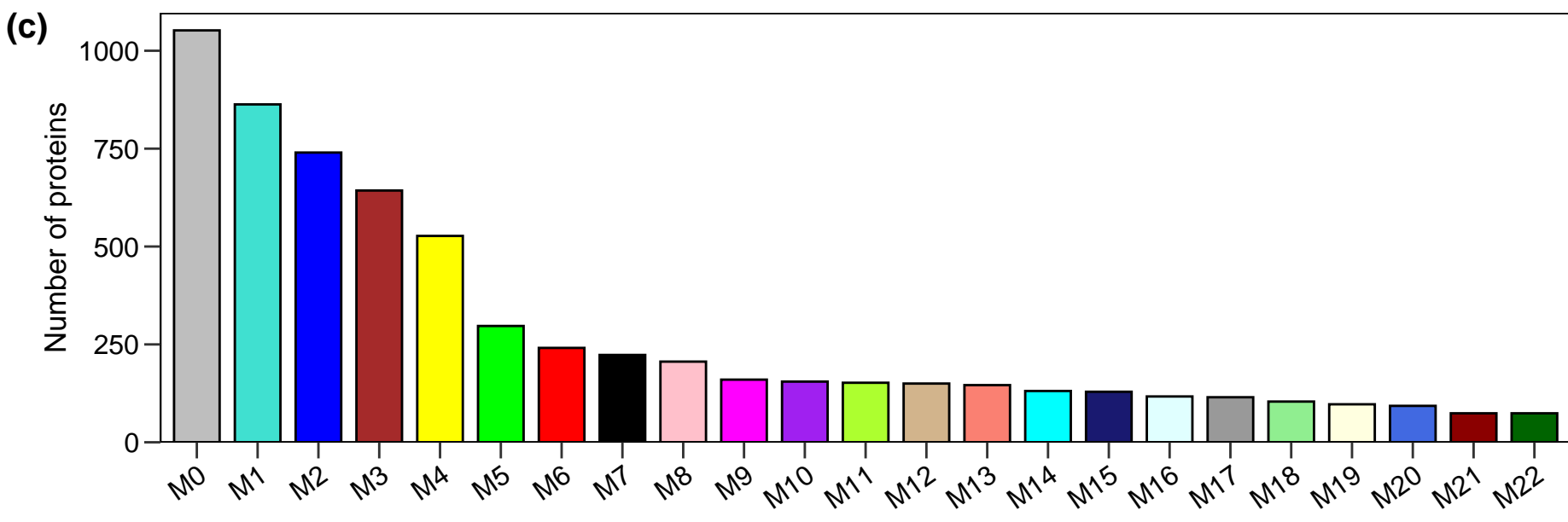

Supplement: Supplementary file 11 — Figure S10. Weighted gene co‐expression network analysis (WGCNA) network construction. (a) Scale‐free fit index (left) and mean connectivity (right) of various soft‐threshold powers. Horizontal dotted line depicts scale‐free fit index of 0.85. Selected soft‐threshold power of 5 is indicated by red. (b) Cluster dendrogram of the proteome using unsupervised hierarchical clustering followed by dynamic tree cutting, which resulted in 22 co‐expressed modules. (c) Number of proteins assigned in each module. Modules were color‐coded and ordered in a descending manner. Module M0 (gray) is for unassigned proteins. [file ACEL-24-e14357-s012.pdf]

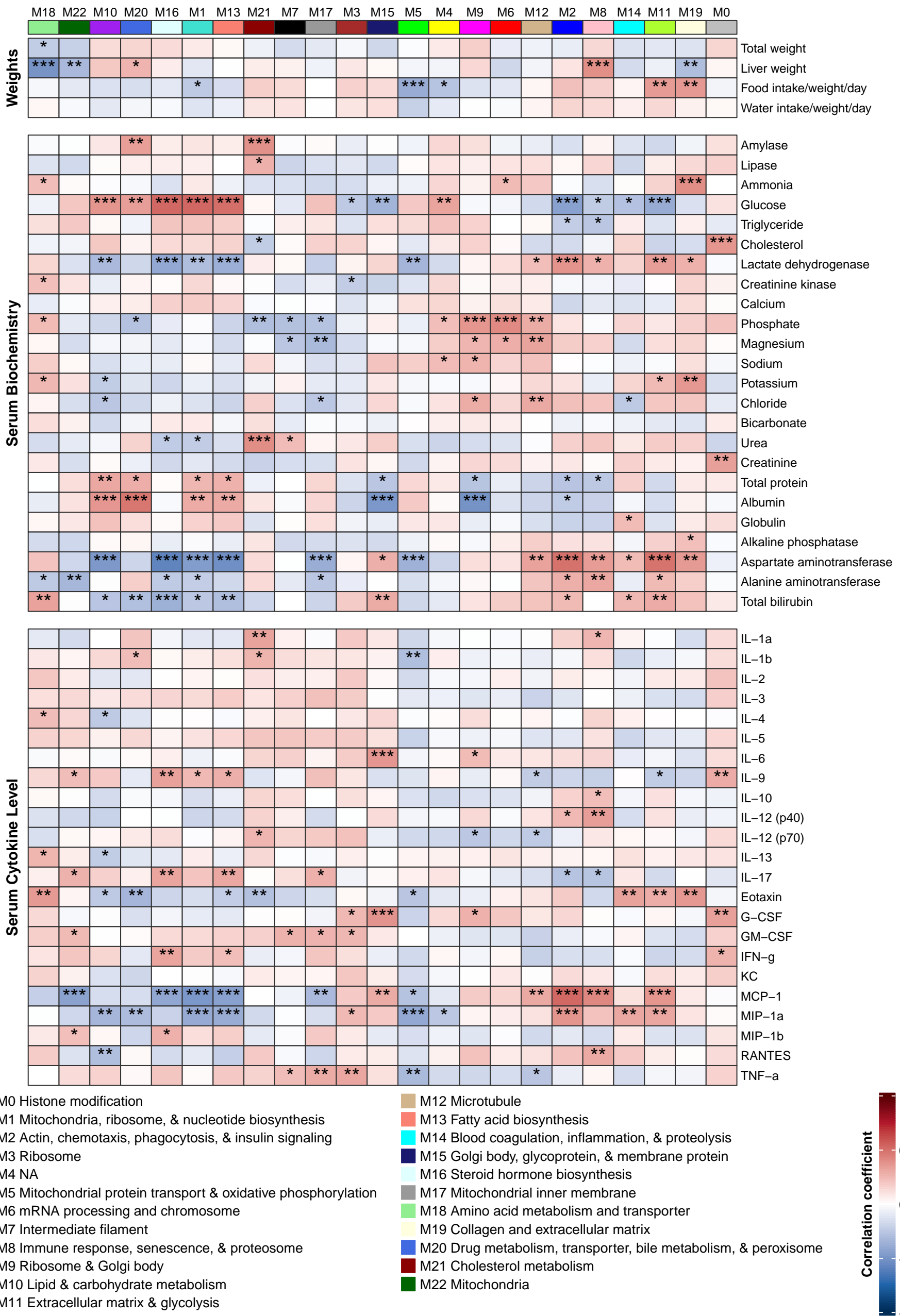

Supplement: Supplementary file 12 — Figure S11. Module–trait relationship analysis of weight, serum biochemistry, and serum cytokine levels. Heatmap summarizing module–trait relationship analysis between the 23 co‐expressed protein modules (columns) and different clinically relevant phenotypes (rows). Degree of positive (red) and negative (blue) correlation are shown, with asterisks representing significance (*p < 0.05, **p < 0.01, ***p < 0.001) based on biweight midcorrelation analysis for weights, serum biochemistry, and serum cytokine levels. Based on GO and KEGG enrichment analysis, selected functional categories of each module were noted at the bottom of the heatmap with their corresponding color codes displayed at the top of the heatmap. [file ACEL-24-e14357-s001.pdf]
