# Supplementary material for: Chronic polypharmacy, monotherapy, and deprescribing: Understanding complex effects on the hepatic proteome of aging mice
Source: Aging Cell. 2024 Oct 27;24(1):e14357. doi: 10.1111/acel.14357 (PMC11709111; doi:10.1111/acel.14357)
Supplement: Supplementary file 7 — Figure S6. Principal component analysis (PCA) comparing deprescribing treatment groups against control and its corresponding chronic drug treatment groups. Samples are color‐coded with corresponding drug treatments and chronic (triangle; solid 95% confidence ellipses) or deprescribed (square; dotted 95% confidence ellipses) drug treatment. The explained variance (%) is noted in brackets beside the axis titles. [file ACEL-24-e14357-s003.pdf]

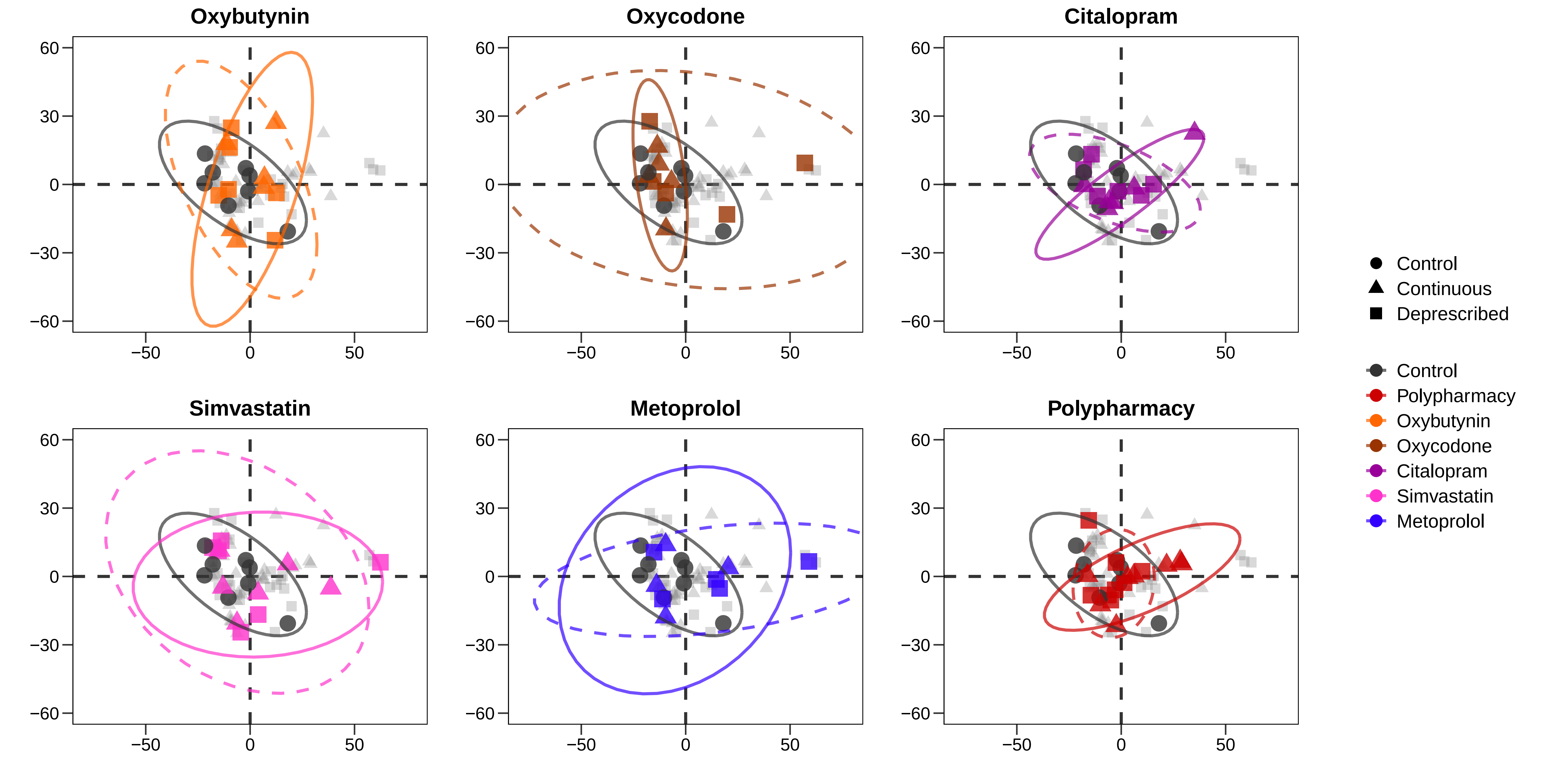

Oxybutynin
Oxycodone
Citalopram
Simvastatin
Metoprolol
Polypharmacy
60
30
0
−30
−60
−50
0
50
Control
Continuous
Deprescribed
Control
Polypharmacy
Oxybutynin
Oxycodone
Citalopram
Simvastatin
Metoprolol
