# Supplementary material for: Chronic polypharmacy, monotherapy, and deprescribing: Understanding complex effects on the hepatic proteome of aging mice
Source: Aging Cell. 2024 Oct 27;24(1):e14357. doi: 10.1111/acel.14357 (PMC11709111; doi:10.1111/acel.14357)
Supplement: Supplementary file 10 — Figure S9. Hepatic transcriptomics of deprescribing polypharmacy. (a) Volcano plot depicting polypharmacy versus polypharmacy deprescribed (left) and polypharmacy deprescribed versus control (right) in the transcriptome level. Vertical lines represent ±1.50 fold change (FC) and the horizontal lines depict raw p < 0.05 and false discovery rate (FDR)‐adjusted p < 0.05. (b) Scatterplot of RNA (y‐axis) and protein (x‐axis) log2(FC) derived from the corresponding pairwise comparisons with Pearson’s correlation analysis conducted on 5967 protein–transcript pairs. Correlation coefficients (R) and p‐values are shown. (c) Pathway‐level comparison as 2D annotation enrichment plot of log2(FC) enrichment score at the RNA (y‐axis) and protein (x‐axis). Gene ontology (GO) terms and Kyoto encyclopedia of genes and genomes (KEGG) pathways are color‐coded, and selected enrichment are highlighted. All enrichment data point depicted are significant enrichment (FDR <0.01) with dot sizes represents p‐value. [file ACEL-24-e14357-s006.pdf]

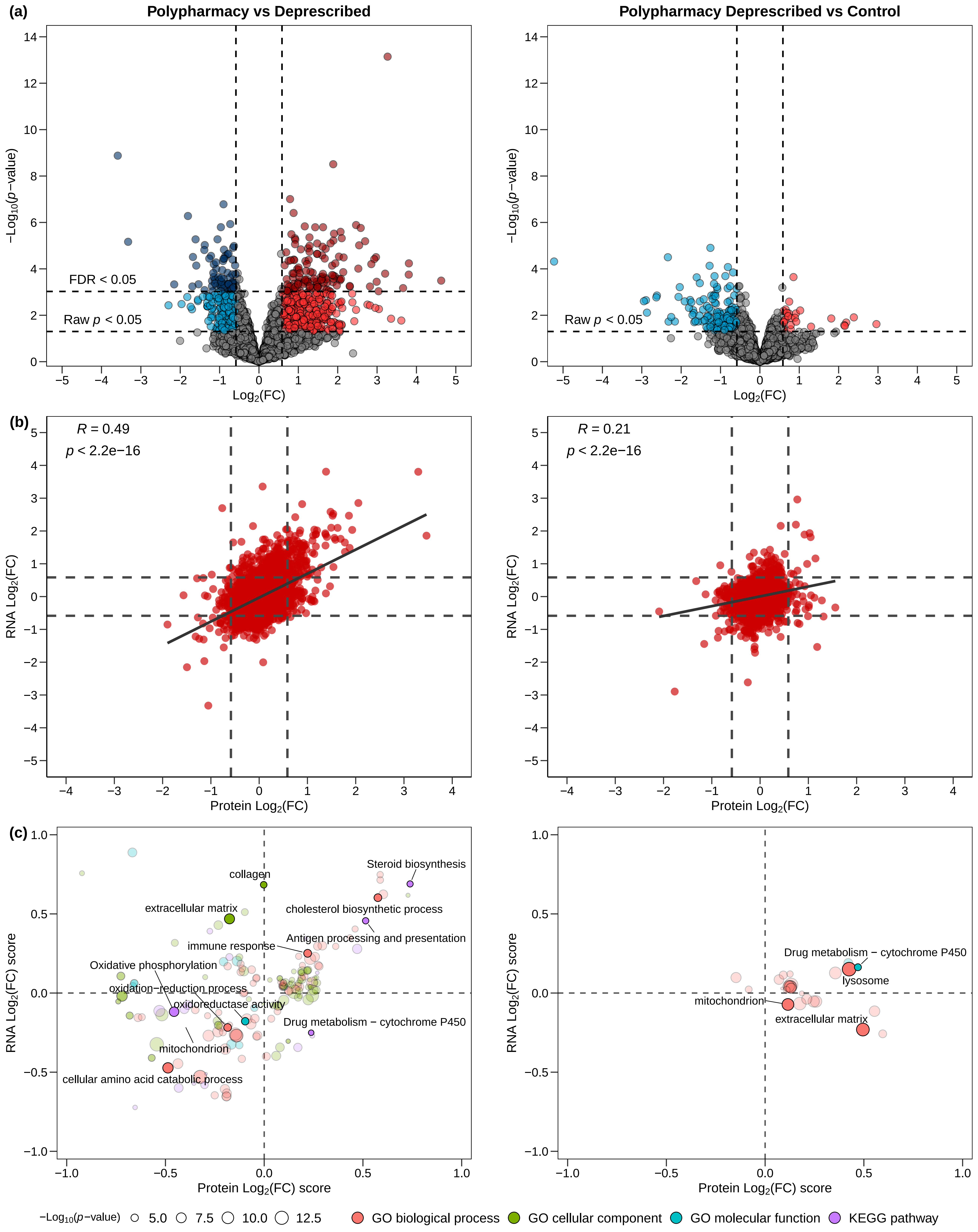

(a)
Polypharmacy vs Deprescribed
Polypharmacy Deprescribed vs Control
FDR < 0.05
Raw p < 0.05
−Log10(p−value)
Log2(FC)
(b)
R = 0.49
p < 2.2e−16
R = 0.21
p < 2.2e−16
RNA Log2(FC)
Protein Log2(FC)
(c)
Steroid biosynthesis
collagen
extracellular matrix
cholesterol biosynthetic process
immune response
Antigen processing and presentation
Oxidative phosphorylation
oxidation−reduction process
oxidoreductase activity
Drug metabolism − cytochrome P450
mitochondrion
cellular amino acid catabolic process
lysosome
RNA Log2(FC) score
Protein Log2(FC) score
−Log10(p−value) 5.0 7.5 10.0 12.5
GO biological process
GO cellular component
GO molecular function
KEGG pathway
