# Supplementary material for: Chronic polypharmacy, monotherapy, and deprescribing: Understanding complex effects on the hepatic proteome of aging mice
Source: Aging Cell. 2024 Oct 27;24(1):e14357. doi: 10.1111/acel.14357 (PMC11709111; doi:10.1111/acel.14357)
Supplement: Supplementary file 13 — Figure S12. Comparing module eigenprotein across treatment groups and expression of hub proteins. Modules of interest were modules that displayed association(s) with any of the geriatric behavioral outcomes, which include (a) M9, (b) M16, and (c) M22. One‐way ANOVA statistics, including degrees of freedom, F statistics, p‐value, and effect size (η 2) were recorded. No statistical significance was detected. (d) Heatmap after hierarchical clustering of the hub proteins across all. Unsupervised hierarchical clustering was conducted with Euclidean distance and complete linkage in which six row and two column clusters were marked. Column is annotated with drug treatment and deprescribing grouping, while row annotation is based on the six co‐expressed modules. Heatmap color code is based on row z‐score scaled with red indicating higher abundance and blue indicating lower. [file ACEL-24-e14357-s011.pdf]

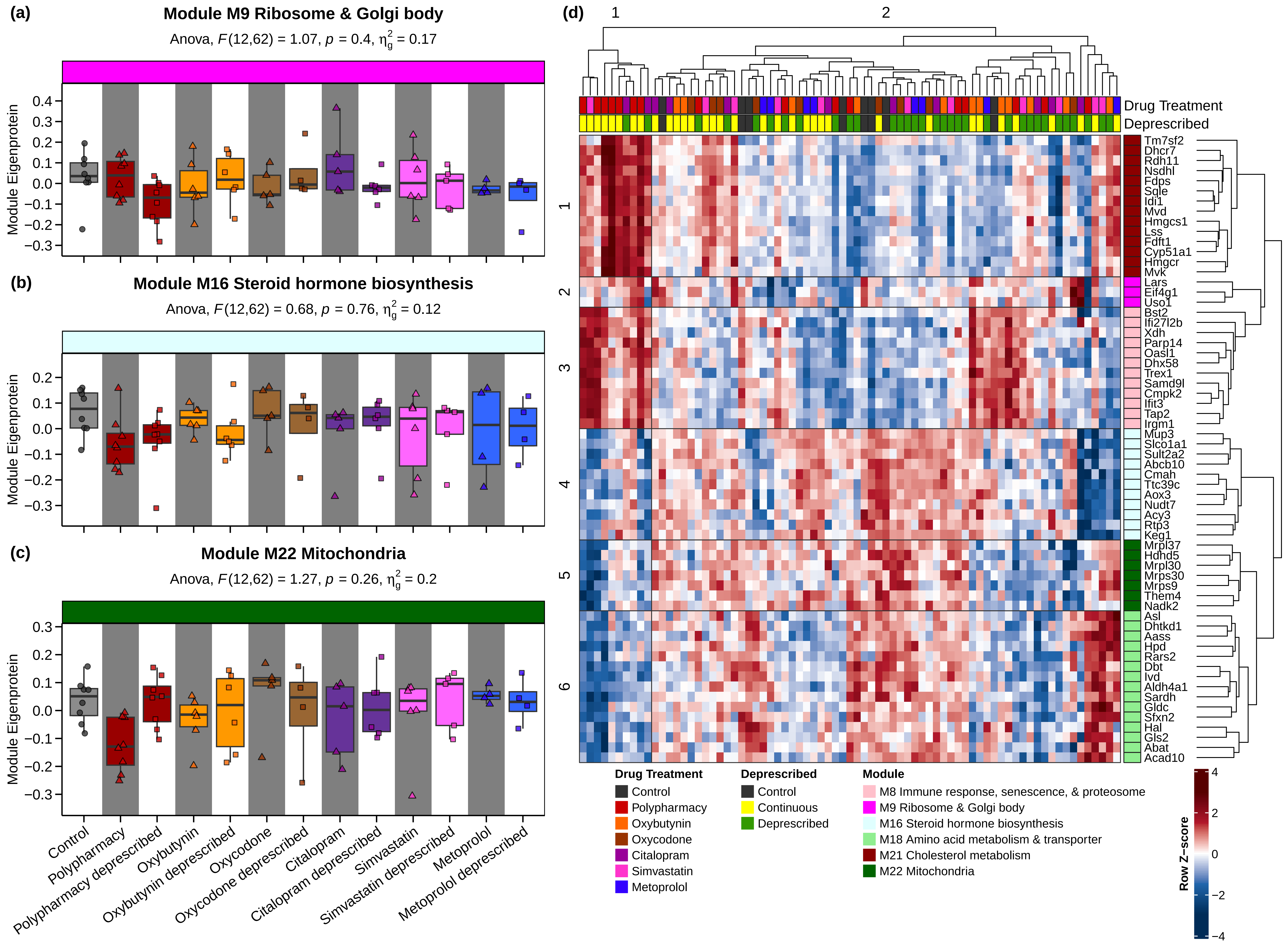
(a)
Module M9 Ribosome & Golgi body
Anova, F(12,62) = 1.07, p = 0.4, η²g = 0.17
Module Eigenprotein
(b)
Module M16 Steroid hormone biosynthesis
Anova, F(12,62) = 0.68, p = 0.76, η²g = 0.12
(c)
Module M22 Mitochondria
Anova, F(12,62) = 1.27, p = 0.26, η²g = 0.2
Control
Polypharmacy
Polypharmacy deprescribed
Oxybutynin
Oxybutynin deprescribed
Oxycodone
Oxycodone deprescribed
Citalopram
Citalopram deprescribed
Simvastatin
Simvastatin deprescribed
Metoprolol
Metoprolol deprescribed
(d)
Drug Treatment
Deprescribed
Tm7sf2
Dhcr7
Rdh11
Nsdhl
Fdps
Sqle
Idi1
Mvd
Hmgcs1
Lss
Fdft1
Cyp51a1
Hmgcr
Mvk
Lars
Eif4g1
Uso1
Bst2
Ifi27l2b
Xdh
Parp14
Oasl1
Dhx58
Trex1
Samd9l
Cmpk2
Ifit3
Tap2
Irgm1
Mup3
Slco1a1
Sult2a2
Abcb10
Cmah
Ttc39c
Aox3
Nudt7
Acy3
Rtp3
Keg1
Mrpl37
Hdhd5
Mrpl30
Mrps30
Mrps9
Them4
Nadk2
Asl
Dhtkd1
Aass
Hpd
Rars2
Dbt
Ivd
Aldh4a1
Sardh
Gldc
Sfxn2
Hal
Gls2
Abat
Acad10
Drug Treatment
Control
Polypharmacy
Oxybutynin
Oxycodone
Citalopram
Simvastatin
Metoprolol
Deprescribed
Control
Continuous
Deprescribed
Module
M8 Immune response, senescence, & proteosome
M9 Ribosome & Golgi body
M16 Steroid hormone biosynthesis
M18 Amino acid metabolism & transporter
M21 Cholesterol metabolism
M22 Mitochondria
Row Z-score
